# TaDIR1-2, a Wheat Ortholog of Lipid Transfer Protein AtDIR1 Contributes to Negative Regulation of Wheat Resistance against *Puccinia striiformis* f. sp. *tritici*

**DOI:** 10.3389/fpls.2017.00521

**Published:** 2017-04-11

**Authors:** Soyed M. Ahmed, Peng Liu, Qinghe Xue, Changan Ji, Tuo Qi, Jia Guo, Jun Guo, Zhensheng Kang

**Affiliations:** State Key Laboratory of Crop Stress Biology for Arid Areas, College of Plant Protection, Northwest A&F UniversityYangling, China

**Keywords:** TaDIR1-2, *Puccinia striiformis* f. sp. *tritici*, wheat, virus-induced gene silencing, reactive oxygen species, salicylic acid

## Abstract

Very few LTPs have been shown to act through plasma membrane receptors or to be involved in the hypersensitive response (HR). DIR1, a new type of plant LTP interacts with lipids *in vitro*, moves to distant tissues during systemic acquired resistance (SAR) and therefore is thought to be involved in long-distance signaling during SAR. However, the exact functions of DIR1 orthologs in cereal species under biotic and abiotic stresses have not been thoroughly defined. In this study, a novel wheat ortholog of the DIR1 gene, TaDIR1-2, was isolated from Suwon11, a Chinese cultivar of wheat and functionally characterized. Phylogenetic analysis indicated that TaDIR1-2 is clustered within the nsLTP-Type II group and shows a closer relationship with DIR1 orthologs from monocots than from eudicots. TaDIR1-2 was localized in the cytoplasm and the cell membrane of wheat mesophyll protoplast. Transcription of TaDIR1-2 was detected in wheat roots, stems and leaves. TaDIR1-2 transcript was significantly induced during the compatible interaction of wheat with the stripe rust pathogen, *Puccinia striiformis* f. sp. *tritici* (Pst). Treatments with salicylic acid (SA) and low temperature significantly up-regulated the expression of TaDIR1-2. Transient overexpression of TaDIR1-2 did not induce cell death or suppress Bax-induced cell death in tobacco leaves. Knocking down the expression of TaDIR1-2 through virus-induced gene silencing increased wheat resistance to Pst accompanied by HR, increased accumulation of H_2_O_2_ and SA, increased expression of TaPR1, TaPR2, TaPAL, and TaNOX, and decreased expression of two reactive oxygen species (ROS) scavenging genes TaCAT and TaSOD. Our results suggest that TaDIR1-2 acts as a negative regulator in wheat resistance to Pst by modulating ROS and/or SA-induced signaling.

## Introduction

Plants encounter various unfavorable conditions, such as the lack of nutrients, water, and light (abiotic stresses) or different types of pathogen attack by bacteria, fungi, and viruses (biotic stresses) (Jones and Dangl, 2006). Plants lack a vertebrate-like circulatory system and antibodies to protect themselves from pathogen attack; instead plants have developed different types of defense mechanisms, including pathogen-associated molecular pattern (PAMP)-triggered immunity (PTI, formerly called basal resistance), effector-triggered immunity (ETI, formerly termed R gene-mediated resistance), and systemic acquired resistance (SAR) (Staskawicz et al., 1995; Jones and Dangl, 2006). PTI is induced when pattern recognition receptors (PRRs) in the plant recognize conserved microbial factors and confers low-level resistance to virulent pathogens. Effectors that trigger ETI are usually perceived by plant resistance proteins (R proteins), which are conserved intracellular receptors of the nucleotide-binding leucine-rich receptor (NB-LRR) class. Effector perception by NB-LRRs is highly specific and can be either direct (with the receptor binding the effector) or indirect (involving accessory proteins). It is reported that ETI triggers a common signaling pathway that leads to accumulation of reactive oxygen species (ROS), rapid ion influxes, followed by salicylic acid (SA) accumulation, expression of pathogenesis-related (PR) genes and hypersensitive response (HR)-related cell death (Cui et al., 2015). In plants, ETI not only induces localized cell death in the infected tissues but also can initiate SAR induced by both avirulent and virulent pathogens to immunize systemic tissues against secondary infections by related or unrelated pathogens (Durrant and Dong, 2004).

The non-specific lipid transfer proteins (nsLTPs) consist of a large, multigene family present in various plant species, with 49 in Arabidopsis, 52 in rice, 156 wheat and 63 members in *Brassica rapa* L. These nsLTPs are characterized by conserved cysteine residues, low molecular mass and a high content of α-helices (Boutrot et al., 2008; Li et al., 2014). A typical nsLTP consists of approximately 100 amino acids and shares an identical motif of 8 cysteine residues, forming 4 intrachain disulfide bridges, with a flexible hydrophobic cavity which interacts non-specifically with lipid molecules (Lee et al., 1998; Samuel et al., 2002). The proteins have traditionally been divided into two different types based on their molecular masses (MM), nsLTP type 1 (nsLTP1, 9 kDa) and nsLTP type 2 (nsLTP2, 7 kDa) (Douliez et al., 2001). Recently, based on sequence similarity and intervals of eight cysteine amino acid residues, Boutrot et al. (2008) proposed a new classification system which categorized nsLTPs into nine types (type I-IX) derived from a genome-wide analysis of rice, wheat and *Arabidopsis thaliana*. Several biological functions have been determined for plant LTPs, including function in cell wall disruption and/or extension (Nieuwland et al., 2005), cuticle synthesis (DeBono et al., 2009) and modulators of plant growth and development (Chae et al., 2009). Interestingly, LTPs are also involved in plant defense reactions against phytopathogens (Salminen et al., 2016). In Arabidopsis, two LTPs, including Defective in Induced Resistance 1 (DIR1), and Azelaic Acid Induced 1 (AZI1), are required for the transmission of a mobile signal(s) during SAR (Maldonado et al., 2002; Jung et al., 2009). The LTP3 contributes to disease susceptibility in Arabidopsis by enhancing abscisic acid (ABA) biosynthesis (Gao et al., 2015). In wheat, a few LTPs have been shown to inhibit growth of the wheat pathogens *Puccinia graminis* f. sp. *tritici, P. triticina* f. sp. *tritici*, and Pyrenophora tritic-repenti (Sun et al., 2008). The wheat TaLTP1.14 is associated with resistance against Fusarium head blight caused by Fusarium graminearum (Schweiger et al., 2013). A wheat LTP1 was shown to bind to a plasma membrane-located receptor for elicitins (Buhot et al., 2001). Overexpression of some LTPs from different plants results in enhanced tolerance to pathogen infection in transgenic Arabidopsis, tobacco and rice (Molina and Garcia-Olmedo, 1997; Jung et al., 2005; Patkar and Chattoo, 2006).

DIR1 is the first genetically characterized LTP participating in long distance signaling during SAR (Maldonado et al., 2002). Structural analysis of Arabidopsis DIR1/LTP indicates that the protein is an atypical LTP2, with a characteristic PxxPxxP (Pro 24 to Pro 30) recognition motif which is unique in DIR1 but not observed in any other LTP2 sequence (Lascombe et al., 2008). In Arabidopsis, the knockout mutant of DIR1 (dir1-1) is compromised in SAR but completely responsive in basal resistance. Based on orthology of the amino acid sequence of DIR1 with those of LTPs and *in vitro* study that showed DIR1 binds 2 lipids (Lascombe et al., 2008), it is suggested that SAR would require the formation of a complex between DIR1 and a lipid molecule which are released on the action of secreted lipases following a pathogen attack (Champigny and Cameron, 2009). A recent study revealed that the DIR1is required for the translocation of G3P to distal tissues during SAR induction in Nicotiana benthamiana plants (Chanda et al., 2011). Further studies supported the importance of DIR1 in the SAR response in dicot plants. In tomato, a putative DIR1 ortholog was identified and shown to be present in petiole exudates from healthy plants, although its' role in SAR was not investigated (Mitton et al., 2009). RNAi-mediated knockdown of two putative DIR1 orthologs from *Nicotiana tabacum* impaired SAR, and transgenic Arabidopsis plants expressing the two DIR1 orthologs from N. tabacum rescued the SAR defect in the Arabidopsis dir1-1 mutant (Liu et al., 2011). Additionally, a DIR1-like protein with high sequence similarity to DIR1 was found in Arabidopsis. Functional analyses indicated that DIR1 and DIR1-like are similarly expressed in healthy and pathogen-challenged plants, and transiently expressed DIR1-like protein complemented the dir1-1 SAR defect (Champigny et al., 2013). Moreover, the dir1-1 mutant occasionally displayed a partially SAR-competent phenotype, suggesting that in some circumstances DIR1-like acts redundantly to DIR1 (Champigny et al., 2013). Orthology analysis and *in vivo* complementation studies provide (Isaacs et al., 2016) evidence that cucumber DIR1 orthologs are functionally equivalent to AtDIR1, indicating the importance of DIR1 in long-distance systemic immune signaling in plants. However, little is known about the roles of DIR1 orthologs in monocot plants, especially in cereal species. Until now, only orthologs of DIR1 in rice (RICE-A and RICE-B) have been functionally characterized (Colebrook, 2010). The results indicated that RICE-A and RICE-B can complement the Arabidopsis dir1-1 mutant, but heterologous expression of AtDIR1, RICE-A, and RICE-B in barley appeared to affect local defense gene expression and symptom development, suggesting the mechanism of induction of acquired resistance differs between Arabidopsis and cereal species.

Stripe rust, caused by the biotrophic fungus Pst, is one of the most important wheat (*Triticum aestivum*) diseases worldwide (Chen, 2005). Deployment of resistant wheat cultivars to control stripe rust is one of the most economic and efficient strategies. Therefore, dissecting molecular mechanisms of interactions between wheat and Pst will facilitate rational use of resistance genes in the improvement of cultivars. In this study, we identified a wheat DIR1 ortholog, designated TaDIR1-2. Transcript profiling of TaDIR1-2 was analyzed in wheat seedlings inoculated with Pst and in plants subjected to environmental stimuli, and the subcellular localization of TaDIR1-2 was determined. Silencing TaDIR1-2 in wheat was performed to analyze whether and how TaDIR1-2 participates in resistance against Pst. Our results indicated that TaDIR1-2 performs a negative role in wheat resistance to stripe rust in a ROS- and/or SA-dependent manner.

## Materials and methods

### Plant and fungal materials, growth conditions, and treatments

Suwon11 (Su11), a Chinese cultivar of wheat and two Pst races CYR23 (incompatible) and CYR31 (compatible) were used to study the wheat-Pst interaction. Su11 carries the resistance gene YrSu conferring resistance to CYR23 but not to CYR31 (Cao et al., 2002). Plant cultivation and Pst inoculation procedures and conditions were followed as described previously by Kang et al. (2002). After inoculation of the first leaves with freshly harvested urediospores of CYR23 and CYR31, leaf tissues were harvested at 0, 12, 24, 48, 72, and 120 h post-inoculation (hpi), and the control plants corresponding to each time point were treated with sterile water. The time points were selected based on the microscopic study of the wheat-Pst interaction (Wang et al., 2007). For chemical treatment assays, 2-week-old wheat seedlings were treated with 2 mM salicylic acid (SA), 100 mM methyl jasmonate (MeJA), 100 mM ethepon (ETH), or 100 mM abscisic acid (ABA) dissolved in 0.1% (v/v) ethanol. Control plants were treated with 0.1% (v/v) ethanol. Leaves of treated and control plants were harvested at 0, 2, 4, 12, and 24 h post-treatment (hpt). For different abiotic stresses, high salinity or drought stress, the roots of 2-week-old wheat seedlings were soaked in 200 mM NaCl (causing osmotic salt stress due to high salinity) or 20% PEG6000 (causing drought stress due to water deficiency), respectively. To assess the effects of wounding, the first leaves were scraped with a sterilized needle. For low-temperature treatment, wheat seedlings were transferred to a 4°C chamber. The first leaves treated with different chemicals and stress treatments along with leaves of the control plants were harvested at 0, 2, 4, 12, and 24 hpt. Intact tissues of different wheat organs from 2-week-old seedlings were collected for tissue-specific expression analysis. All freshly collected samples were rapidly frozen in liquid nitrogen and stored at −80°C until the extraction of total RNA or DNA. For each time point, three independent biological replications were performed.

### RNA/DNA isolation and cDNA synthesis

The DNeasy Plant Mini Kit (Qiagen) was used for extraction of Genomic DNA from wheat leaves. Total RNA from wheat leaves that ware challenged with chemicals, abiotic stresses elicitors, Pst as well as different wheat tissues were extracted with the Trizol TM Reagent (Invitrogen, Carlsbad, CA, USA) following the manufacturer's instructions. Contaminating DNA was removed by treatment with DNase I. First strand cDNA was synthesized using 2 μg of total RNA with the RT-PCR system (Promega, Madison, WI, USA) and Oligo (dT)18 primer.

### Cloning, identification and sequence analysis of *TaDIR1-2*

PCR was performed with the primers TaDIR1-2(ORF)-S and TaDIR1-2(ORF)-AS (Supplementary Table S1) designed based on the sequences of DIR1-2 orthologous group (Supplementary Figure S1) to amplify TaDIR1-2. The amplified product was cloned into the pGEM-T Easy Vector (Promega, Madison, WI, USA) for sequencing. This cloned sequence was aligned with the wheat cv. Chinese Spring genome, based on the data of International Wheat Genome Sequencing Consortium (https://urgi.versailles.inra.fr/blast/). The chromosomal location and predicted related sequences were also obtained from this website. ORF Finder and the BLAST (https://www.ncbi.nlm.nih.gov/). programs ware used for the analyses of cDNA and amino acid sequences. Conserved domains were identified using Pfam (http://pfam.xfam.org/). and Inter ProScan (http://www.ebi.ac.uk/interpro/search/sequence-search). Multiple sequence alignment was performed with DNAMAN software (Lynnon Biosoft, USA), and Mega 5.0 software was used for phylogenetic tree construction (Tamura et al., 2011). si-Fi software v1.4.0 (http://labtools.ipk-gatersleben.de/) was used to identify off-target sequence.

### Quantitative reverse transcription PCR (qRT-PCR) analysis

Specific primers were designed (Supplementary Table S1) and used as described previously (Duan et al., 2011). The ABI PRISM 7500 software tool (Applied Biosystems, Foster City, CA, USA) was used to generate threshold values (CT) for the quantification of relative gene expression using the comparative 2^−ΔΔCt^ method (Livak and Schmittgen, 2001) and the data were normalized against expression of the wheat elongation factor TaEF-1α gene (GenBank accession no. Q03033) (Paolacci et al., 2009). All reactions were performed in triplicate. To ensure specific amplification, dissociation curves were generated for each reaction. The qRT-PCR analysis for respective experiment was replicated three times with similar results.

### Subcellular localization of TaDIR1-2::GFP fusion protein

For subcellular localization in wheat protoplasts, the TaDIR1-2 protein-encoding sequence was amplified and inserted into the PstI and XbaI sites of the pCaMV35S::GFP vector to generate the pCaMV35S::TaDIR1-2::GFP fusion vector by PCR using primers TaDIR1-2(163)-S and TaDIR1-2(163)-AS (Supplementary Table S1). The protoplasts were isolated from mesophyll tissue of 8–10-day-old wheat seedlings as previously described (Li et al., 2015). The fusion pCaMV35S::TaDIR1-2::GFP construct and the control plasmid pCaMV35S::GFP were transformed into wheat protoplasts by the PEG-mediated transformation system (Bio-Rad, Hercules, CA, USA), and then the PEG-transfected mesophyll protoplasts were submersed in W5 solution and incubated at 23°C for 18 h in a dark chamber. Fluorescent signals were observed with a Zeiss LSM510 confocal laser microscope (Zeiss, Germany) with a 480-nm filter as previously described (Ito and Shinozaki, 2002).

### Transient overexpression of *TaDIR1-2* in tobacco (*Nicotiana benthamiana*)

The recombinant vectors, PVX-TaDIR1-2, PVX-pBin19, PVX-Bax, PVX-Avr1b, and PVX-eGFP were constructed by inserting the protein-encoding sequence into the ClaI and SalI sites using the respective primers (Supplementary Table S1) and transformed individually into Agrobacterium tumefaciens strain GV3101 as described previously (Dou et al., 2008). A. tumefaciens cell suspensions carrying the respective plasmids were cultured in 5 ml of LB medium containing kanamycin, rifampicin and gentamycin, and then collected at an OD600 of 0.6–1.2 and re-suspended in 10 mM MgCl2 to a final density of 0.2–0.3 at OD600. The cells were infiltrated into 4–6-week-old tobacco (Nicotiana benthamiana) leaves as described by Wang et al. (2011). The same infiltration site was challenged with the Bax gene carrying A. tumefaciens cell suspensions at 16 h after f the 1st infiltration. PVX-empty vector (EV) and PVX-Avr1b were infiltrated as negative and positive controls, respectively. Subsequently, PVX-pBin19 was used to suppress gene silencing of PVX-TaDIR1-2 (Voinnet et al., 2003), and PVX-eGFP was used to assess the efficiency of the experiments. Green fluorescence was identified in eGFP-treated leaves at 4 days after the 2nd infiltration. Symptom development was examined and photos were taken 4 days after the 2nd infiltration. The experiment was replicated three times with similar results.

### BSMV-mediated *TaDIR1-2* gene silencing

A small fragment with 155-bp was used to silence TaDIR1-2. The fragment of TaDIR1-2 with NotI and PacI restriction sites was obtained by reverse transcription PCR to modify the original BSMV:γ vector. Capped *in vitro* transcripts of BSMV RNAs were prepared from the linearized plasmids γ-TaPDS-as, γ-TaDIR1-2, γ, α, β using a Message T7 *in vitro* transcription kit (Ambion, Austin, TX) according to the manufacturer's protocol. During the inoculation, the RNA transcripts were diluted 4-fold, and 2.5 μL of each transcript (BSMV RNA α, β, γ; γ-TaPDS and γ-TaDIR1-2) were mixed with 42.5 μL of FES buffer (Pogue et al., 1998). The mixture was inoculated individually onto the second leaf of 2-leaf wheat seedlings by gently rubbing the leaf surface with a gloved finger as described previously (Scofield et al., 2005). The virus-infected wheat seedlings were incubated for 24 h in darkness and high humidity, and then incubated in a growth chamber at 25 ± 2°C with a 16-h photoperiod. In total, 40 seedlings were inoculated with each of the three viruses (BSMV:γ, BSMV:TaPDSas and BSMV:TaDIR1-2 as). In addition, 40 seedlings were treated with FES buffer as the control. To check the BSMV infection, BSMV:γ and BSMV: TaPDS were used as controls. After 10 dpi, the fourth leaves of BSMV-infected seedlings were further inoculated with freshly harvested urediospores of Pst race CYR23 or CYR31, and the Pst infected plants were consequently maintained in the condition described above. After 15 dpi, the infection types of Pst were examined on the basis of McNeal measurements scale (McNeal et al., 1971) and photos were taken. Three independent sets of plants were prepared for each assay. The Pst-infected fourth leaves were collected at 0, 24, 48, and 120 hpi for histological observation as well as RNA isolation. The silencing efficiency of TaDIR1-2 knockdown plants and the relative transcript levels of the pathogenesis related (PR) protein genes TaPR1 and TaPR2, asecondary metabolite gene TaPAL, and ROS related genes, including superoxide dismutase (TaSOD), catalase (TaCAT) and NADH-oxidase (TaNOX), were analyzed by qRT-PCR in comparison with the control plants in each assay as described above. The primers were used to perform qRT-PCR are listed in Supplementary Table S1.

### Histological observation of fungal development in *TaDIR1-2*-knockdown plants

The fungal development in TaDIR1-2-knockdown plants or control plants challenged with Pst was examined microscopically. For histological observation, collected leaf samples were cut into 1.5 cm long segments and treated with ethanol: acetic acid (1:1 v/v) for decolorization, and saturated chloral hydrate was used to clarify the leaf tissue. To examine the necrotic cell area, the auto-fluorescence of the pathogen-attacked necrotic cells was observed under a fluorescence microscope (excitation filter 485 nm, dichromic mirror 510 nm, and barrier filter 520 nm) and the necrotic area was measured using DP-BSW software. To measure the area of H_2_O_2_ accumulation in the infection sites, leaf samples were stained with 3,3′-diamino benzidine (DAB; Amresco, Solon, OH, USA) as previously described (Wang et al., 2007). H_2_O_2_ accumulation was observed under differential interference contrast optics and the area was measured with DP-BSW software. The hyphal length, haustoria, haustorial mother cell and infection area were determined under an Olympus BX-53 microscope (Olympus Corp., Tokyo) and the length and area were calculated with DP-BSW software. According to Bozkurt et al. (2010), after entering the Pst urediniospores in the plant, a substomatal vesicle forms within the stomatal cavity, from which three infection hyphae grow, an infection peg develops and breaching the mesophyll cell wall, were considered as successful penetrations and were microscopically examined to assess the infection hyphae, haustoria, haustorial mother cell and infection area. For each treatment, 50 infection sites were examined. Standard deviations and Student's *t*-test were used for statistical analysis.

### SA quantification in *TaDIR1-2*-knockdown plants

For the quantification of SA, approximately 100–200 mg of fresh leaf tissue of each sample was used to extract SA, and analyzed with HPLC-MS (API 2000; AB SCIEX, Framingham, USA) by the protocol previously described (Segarra et al., 2006).

### Statistical analyses

Microsoft Excel software was used to calculate mean values and standard errors. A one-way analysis of variance (ANOVA) was performed using the SPSS 16.0 (SPSS Inc., Chicago, Illinois, USA) statistical software to determine the significant differences between control and treatment or between time-course points. The probability (*P*) value < 0.05 was used to measure the significant change with unequal variance.

## Results

### Identification of wheat orthologs of AtDIR1 and OsDIR1

To systematically identify DIR1 orthologs from *T*. *aestivum*, Aegilops tauschii (A. tauschii) and *Triticum urartu* (*T. urartu*), a genome-wide search using DIR1 protein sequences from Arabidopsis and rice as query revealed a total of 25 putative DIR1 loci in wheat (3, 7, and 15 were obtained from the Aegilops tauschii, *Triticum urartu* and *Triticum aestivum* genome databases, respectively). Out of 15 putative DIR1 protein sequences from the *Triticum aestivum* genome database, loci 5, 8, and 2 were located in sub-genomes A, B, and D, respectively. To better understand the evolutionary relationship of putative DIR1 orthologs in wheat with other plant DIR1 proteins, a phylogenetic tree of wheat, rice, Arabidopsis DIR1 proteins was constructed based on the full-length amino acid sequences (Supplementary Figure S1). The resulting dendrogram indicated that putative DIR1 orthologs from wheat were clustered into six major groups, including DIR1-1, DIR1-2, DIR1-3, DIR1-4, DIR1-5, and DIR1-6. DIR1-1 orthologs showed highest orthology with AtDIR1 in Arabidopsis and OsDIR1-A (RICE-A) in rice. However, the DIR1 orthologs in the other five groups showed much higher similarity with OsDIR1-B (RICE-B) than OsDIR1-A, AtDIR1, and AtDIR1-like.

### Cloning and sequence analysis of *TaDIR1-2*

**Figure 1:**
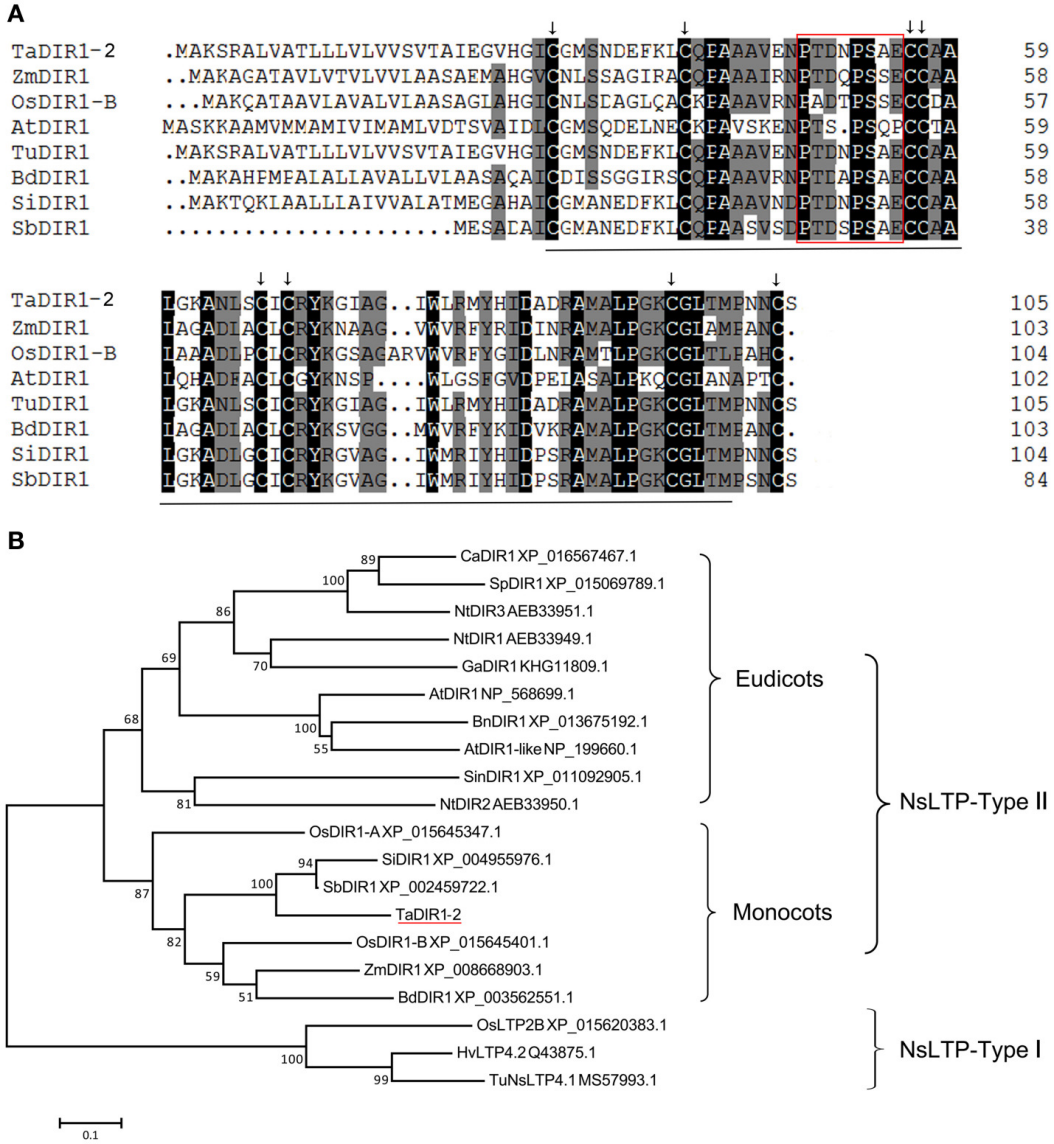
**Multiple alignment and phylogenetic analysis of the deduced amino acid sequences of TaDIR1-2 with other DIR1 and lipid transfer proteins (LTPs) from higher plants. (A)** Sequence alignment of AtDIR1 and its Orthologs from monocot plants. The black line indicates the nsLTP (Non-specific lipid-transfer protein) domain, the black arrow represents the 8-cysteine signature indicating the LTP type 2 family, and the red box indicates a proline-rich domain. **(B)** A representative phylogenetic tree of TaDIR1-2 with other DIR1 proteins and LTPs from higher plants. At, *Arabidopsis thaliana*; Bd, *Brachypodium distachyon*; Bn, *Brassica napus*; Ca, *Capsicum annuum*; Ga, *Gossypium arboreum*; Hv, *Hordeum vulgare* subsp. Vulgare; Nt, *Nicotiana tabacum*; Os, *Oryza sativa*; Sb, *Sorghum bicolor*; Si, *Setaria italic*; Sin, *Sesamum indicum*; Sp, *Solanum pennellii*; Ta, *Triticum aestivum*; Tu, *Triticum urartu*; Zm, *Zea mays*. Bootstrap values (>50%) based on 1000 replications are shown at branch nodes. NsLTP-Type-I was used as an out-group. The bar indicates 1 substitution per 10 nucleotide positions.

According to DIR1 sequences identified from the wheat genome, we designed primers and cloned one DIR1 orthologous gene in the DIR1-2 group from wheat cv. Su11, designated TaDIR1-2. Sequence alignment indicated that TaDIR1-2 has high identity with DIR1 proteins from monocot plants, 57–99% (60–94% nucleotide) with other DIR1 orthologs in wheat (Supplementary Figure S2), 66% (60% nucleotide) with rice OsDIR1-B and 60% (64% nucleotide) with Brachypodium BdDIR1, respectively. In contrast, TaDIR1-2 showed only 45% identity with Arabidopsis AtDIR1. The protein conserved domain analysis showed that TaDIR1-2 contains a nsLTP region from 29 to 100 amino acids residues, an eight cysteine signature with a central motif (C68-X-C70), and a proline rich domain (Figure 1A).

To study the evolutionary relationships of DIR1 orthologs, putative DIR1 orthologous proteins from eudicots and monocots plants, respectively, were selected to construct a maximum likelihood tree. The result showed that the putative DIR1 from different organisms formed a monophyletic group, NsLTP-Type II containing monocot and eudicot subgroups. OsLTP2B, TuNSLTP, and HvLTP4.2 were clustered into NsLTP-Type I as an out group (Figure 1B). TaDIR1-2 that clustered in the monocot subgroup showed closer relationships with SbDIR1, SiDIR1, ZmDIR1, BdDIR1, and OsDIR1-B than OsDIR1-A (Figure 1B).

### TaDIR1-2 is localized in the cytoplasm of wheat cells

**Figure 2:**
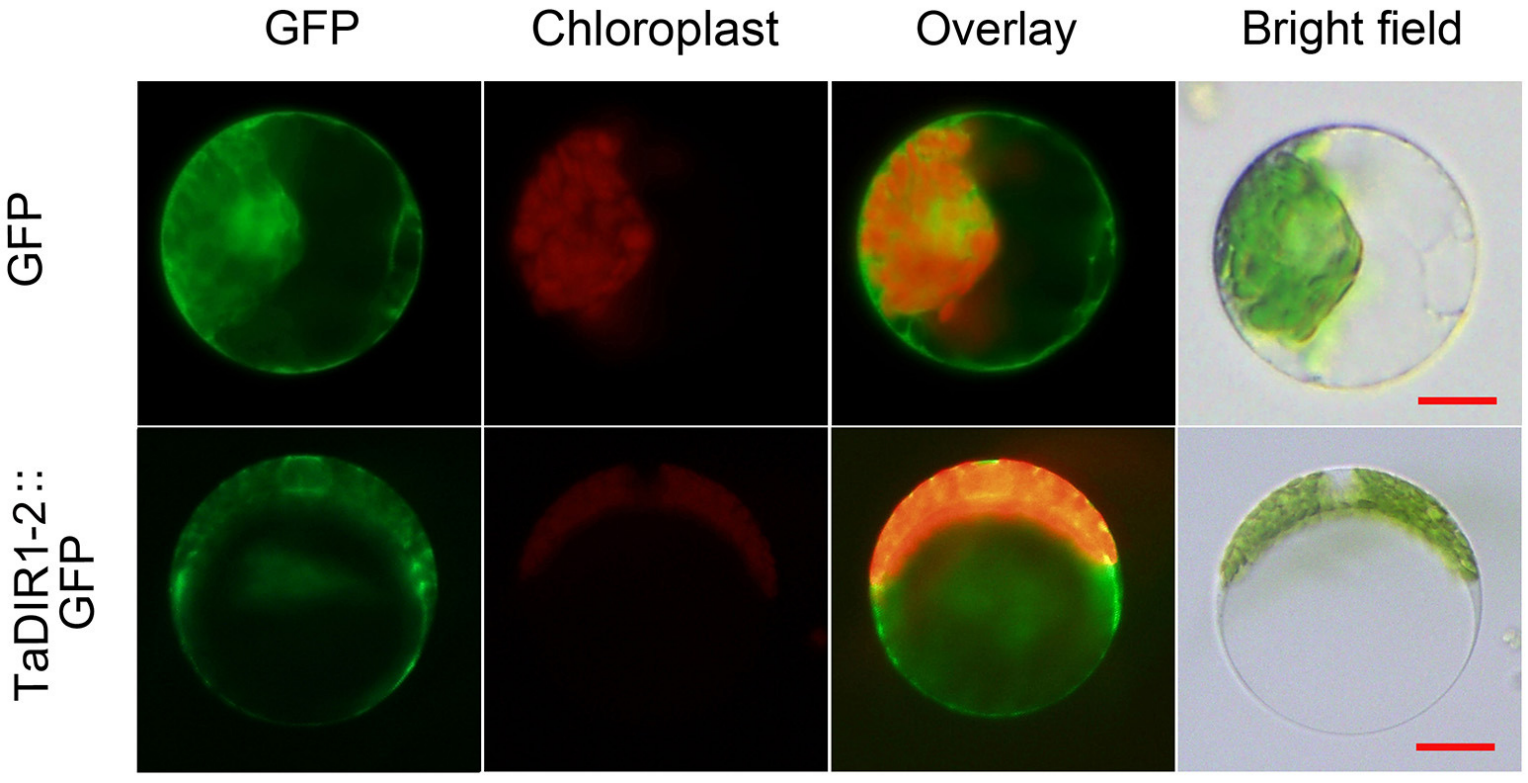
**Subcellular localization of TaDIR1-2 protein in wheat protoplasts**. Green fluorescent protein (GFP), TaDIR1-2-GFP fusion protein and chloroplast autofluorescence signals were observed under a confocal microscope and imaged using a 488-laser excitation light source. Bright field images show the equivalent field observed under white light. Bar, 20 μm.

In Arabidopsis LTP3 protein was localized to the cytoplasm (Guo L. et al., 2013). In wheat TaLTP3 protein was shown to be localized in the cell membrane and cytoplasm of tobacco epidermal cells (Wang et al., 2014). In this study, subcellular localization of TaDIR1-2 in wheat cells was determined by the transfection of recombinant pCaMV35S::TaDIR1-2::eGFP into mesophyll protoplasts, and the empty pCaMV35S::eGFP vector was used as the control. By laser-scanning confocal microscopy, the green fluorescence of fusion eGFP was detected throughout the cell, including in the nucleus. In contrast, pCaMV35S::TaDIR1-2::GFP fusion proteins were localized in the cytoplasm, perhaps in the cell membrane, of wheat mesophyll protoplasts (Figure 2).

### *TaDIR1-2* expression is detected in different wheat tissues

**Figure 3:**
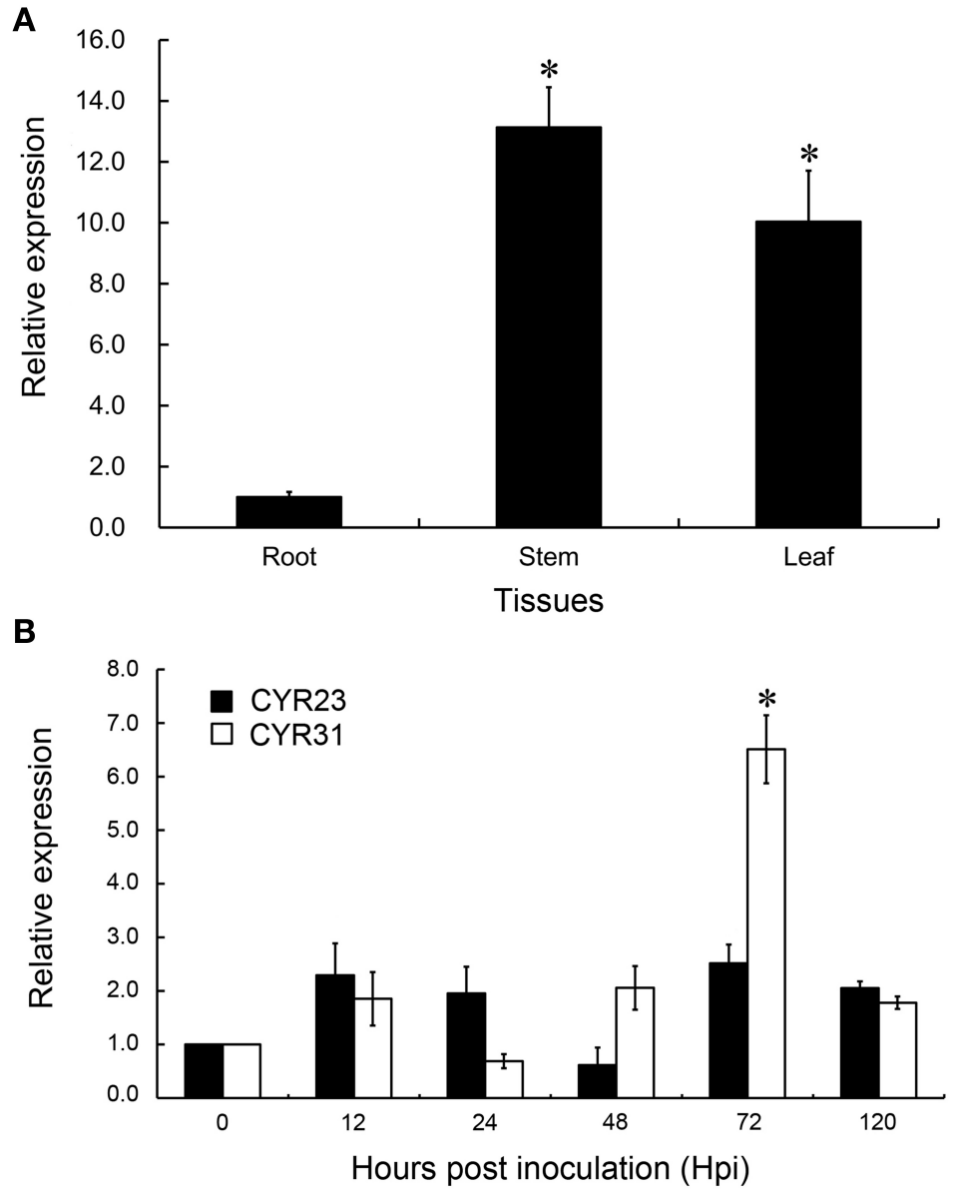
**Transcript profiles of TaDIR1-2 in different wheat tissues and in wheat leaves in response to virulent and avirulent Pst races (A)** Samples were collected from leaves, stems and roots. **(B)** Wheat leaves were sampled for CYR23- and CYR31- inoculated and mock-inoculated plants at 12, 24, 48, 72, and 120 hpi and, additionally, the mock-inoculated at 0 hpi. Results are shown as means ± standard deviation of three biological replications. Relative gene quantification was calculated by the comparative ΔΔCt method. Data were normalized to the expression level of the wheat elongation factor TaEF-1α (GenBank accession no. Q03033). Asterisks indicate a significant difference (*P* < 0.05) from the root **(A)** and from 0 hpi **(B)** by Student's *t*-test.

In Arabidopsis, DIR1 is expressed constitutively to low levels in all the tissues tested including seedlings, roots and flowers and in the living cells of leaves (Champigny et al., 2011). To examine the expression patterns of TaDIR1-2, the transcript level of TaDIR1-2 in different tissues of 2 weeks old wheat plants was measured by qRT-PCR analysis. The results revealed that TaDIR1-2 transcripts were detectable in all tested wheat tissues (root, stem and leaf) and the abundance of TaDIR1-2 transcripts in stem and leaf were significantly higher than in root (Figure 3A).

### *TaDIR1-2* expression is significantly increased upon virulent *Pst* infection

During infection with Pst, TaDIR1-2 expression was induced to different extents in compatible (CYR31) and incompatible (CYR23) interactions (Figure 3B). During the compatible interaction, theTaDIR1-2 transcript level was induced at all-time points except at 24 hpi, when its transcript level slightly decreased. At 72 hpi, TaDIR1-2 transcripts reached a peak which was 6.5-fold higher than that in the control plants. However, in the incompatible interaction, no significant up- or down-regulation (*P* < 0.05) for TaDIR1-2 transcript level was observed (Figure 3B). Thus, it is reasonable to assume that TaDIR1-2 may contribute to negative regulation of wheat resistance against Pst infection. To support this supposition, further experiments were performed subsequently.

### *TaDIR1-2* is induced under SA and low temperature treatments

**Figure 4:**
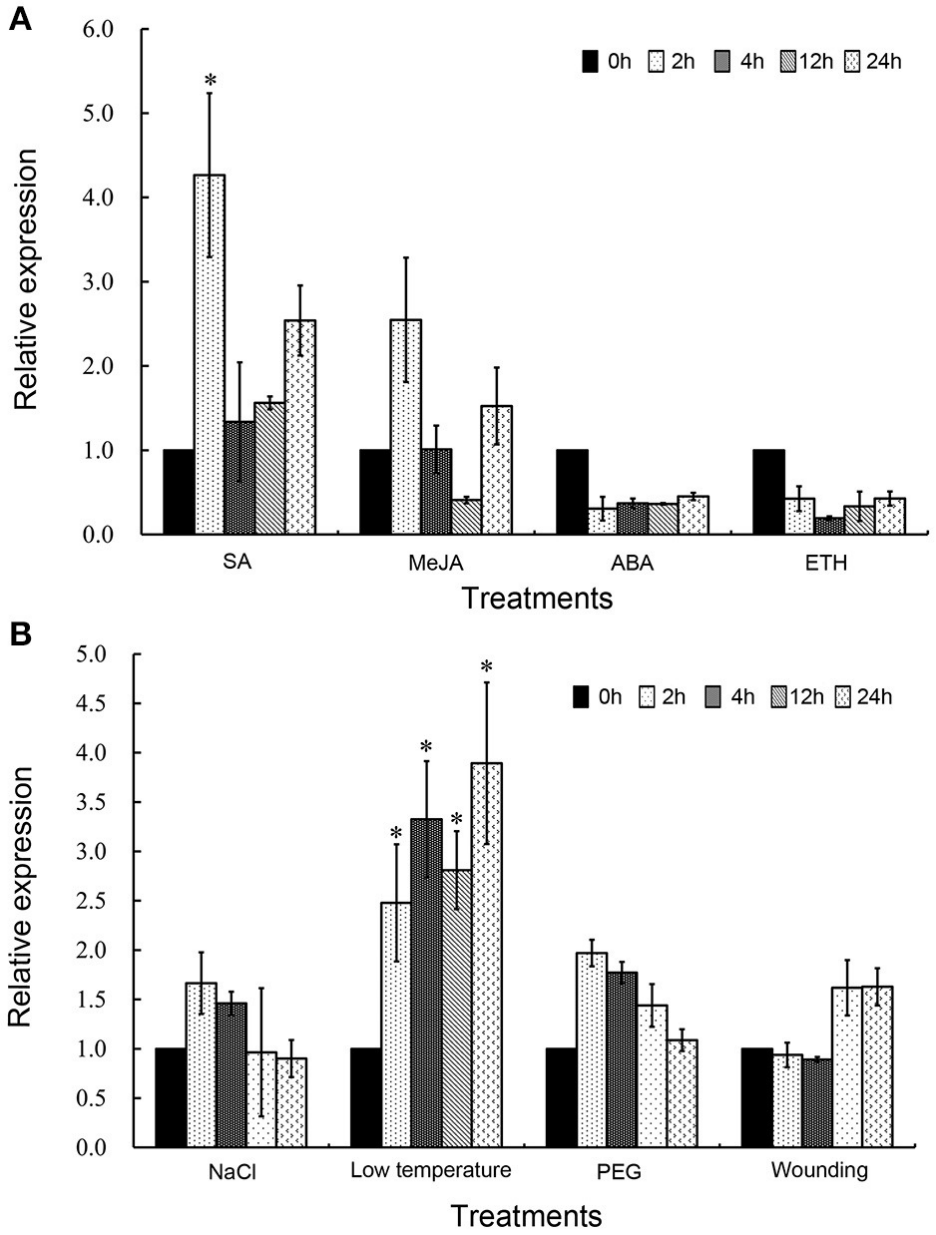
**Transcript profiles of TaDIR1-2 in response to exogenous hormones and abiotic stresses. (A)** Exogenous hormones: SA, salicylic acid; MeJA, methyl jasmonate; ABA, abscisic acid; ETH, ethylene. **(B)** Abiotic stresses: High salinity (200 mM NaCl); low temperature (4◦C); drought (20% PEG6000); wounding (cutting wheat leaves). The mock control was treated with 0.1% (v/v) ethanol solution. Results were shown as means ± standard deviation of three biological replications. Relative gene quantification was calculated by the comparative ΔΔCt method. Data were normalized to the expression level of the wheat elongation factor TaEF-1α (GenBank accession no. Q03033). Asterisks indicate a significant difference (*P* < 0.05) from the 0 hpt using Student's *t*-test.

To determine whether the transcription of TaDIR1-2 was affected by exogenous hormone elicitors and abiotic stresses, we performed the qRT-PCR analysis. As shown in Figure 4A, TaDIR1-2 was mainly induced by SA treatment but showed no significant response to the other treatments. In the SA treatment, the expression of TaDIR1-2 was increased at all-time points and peaked at 2 hpt with 4.5-fold expression. These results suggested that TaDIR1-2 may function in the SA-dependent signaling pathway.

For abiotic stresses, wheat seedlings were treated with NaCl, low temperature (4°C), PEG6000 (Khodarahmpour, 2011) and wounding. The results showed that the low-temperature (4°C) significantly up-regulated the expression of TaDIR1-2. In contrast, salt stress, drought stress, or wounding did not cause any significant changes in the expression of TaDIR1-2 (Figure 4B).

### TaDIR1-2 alone can not induce cell death or suppress bax-induced programmed cell death (PCD)

**Figure 5:**
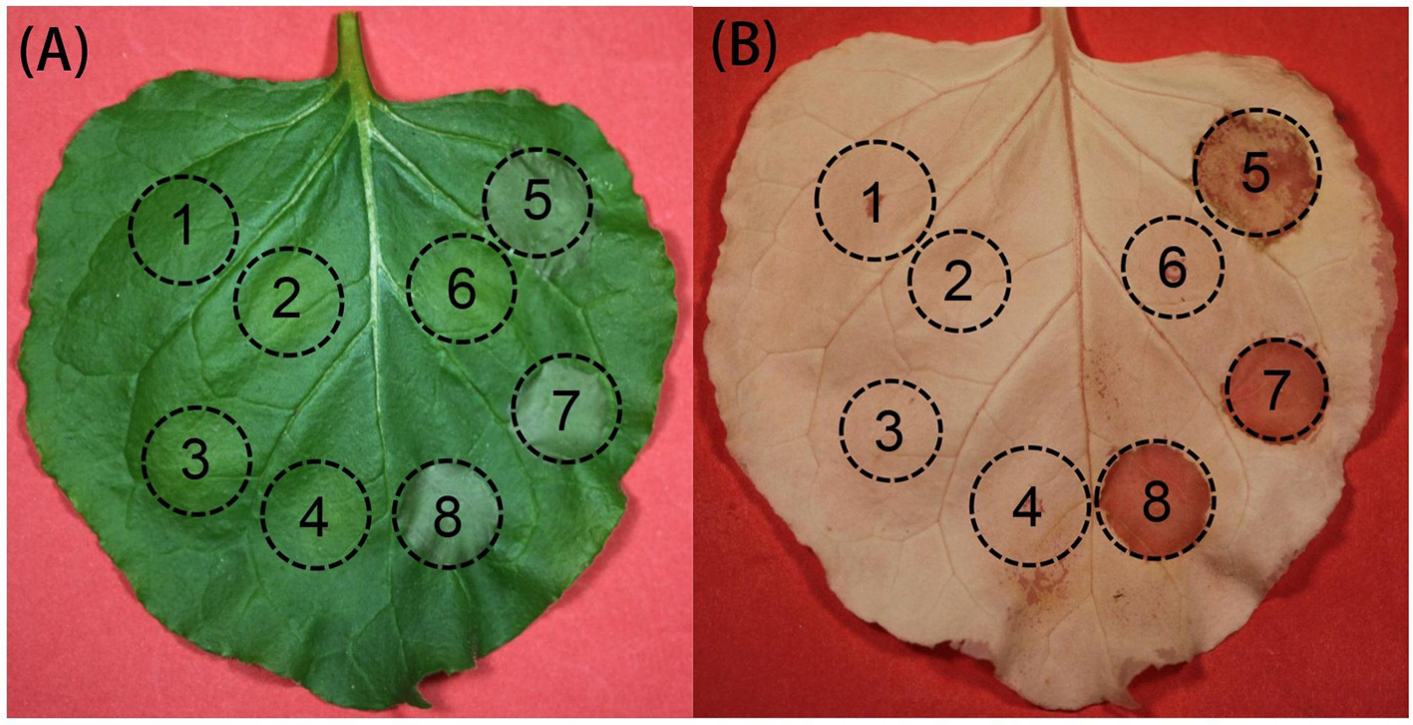
**Transient overexpression of TaDIR1-2 in tobacco (Nicotiana benthamiana). (A)** Tobacco leaves were infiltrated with Agrobacterium tumefaciens cells carrying empty vector (EV), Avr1b, (TaDIR1-2+pBin19) or eGFP alone (circles 1-4), or followed 16 h later with a 2nd infiltration of **(A)**. Tumefaciens cells carrying Bax (circles 5-8). Photos were taken 4 days after the 2nd infiltration. **(B)** The same leaves collected at the same time (4 days after 2nd infiltration) and submerged for 7 days in 100% ethanol when photos were taken. Circles: 1, empty vector (EV); 2, Avr1b; 3, (TaDIR1-2+pBin19); 4, eGFP; 5, empty vector (EV)+Bax; 6, Avr1b+Bax; 7, (TaDIR1-2+pBin19)+Bax; 8, eGFP+Bax. Circles indicate infiltrated areas; same position represents same sample.

PCD is an important element for plant immunity against biotic and abiotic stress as well as for plant development and proliferation (Gadjev et al., 2008), including vacuolar cell death, hypersensitive cell death and necrosis which is induced by abiotic stresses through mitochondrial dysfunction that include uncoupling of respiration, the production of ROS and RNS, a drop in ATP level and mitochondrial membrane permeabilization (Van Doorn et al., 2011). Members of the diverse BAX family proteins in animals, have emerged as important regulators of PCD, which act as either activators or suppressors of PCD in animal as well as in transgenic plants (Kroemer, 1997; Mitsuhara et al., 1999). VAD1 (vascular associated death1), containing a lipid binding signaling domain and ACD1 (accelerated cell death1) gene encoding a protein homologous to a mammalian glycolipid transfer protein (GLTP), are expected to control the cell-to-cell propagation of PCD in Arabidopsis (Brodersen et al., 2002; Lorrain et al., 2004). To determine whether TaDIR1-2 could induce cell death or suppress PCD induced by the pro-apoptotic protein BAX, TaDIR1-2 was transiently overexpressed in tobacco leaves using potato virus X (PVX) delivery in combination with the Bax system by an A. tumefaciens-mediated transient overexpression assay. Agrobacterium strains carrying the recombinant genes were infiltrated into N. benthamiana leaves 16 h prior to infiltration with Bax-carrying strains. The green fluorescence was detected at 4 dpi of 2nd infiltration with eGFP treatment (Supplementary Figure S3). When leaves were infiltrated with empty vector (EV), Avr1b, (TaDIR1-2+pBin19) or eGFP, no cell death phenotype was observed (Figures 5A,B; circles 1, 2, 3, and 4). However, tobacco leaves infiltrated with EV+Bax, (TaDIR1-2+p19)+Bax or eGFP+Bax (Figures 5A,B; circles 5, 7, and 8) showed the Bax-triggered PCD phenotype at 4 dpi of 2nd infiltration. In addition, leaves infiltrated with Avr1b+BAX, suppressed the Bax-induced PCD and no cell death symptoms appeared (Figure 5; circle 6). These results indicated that TaDIR1-2 by itself is unable to induce PCD or suppress Bax-triggered PCD.

### Increased wheat resistance to *Pst* in *TaDIR1-2*-knockdown plants

**Figure 6:**
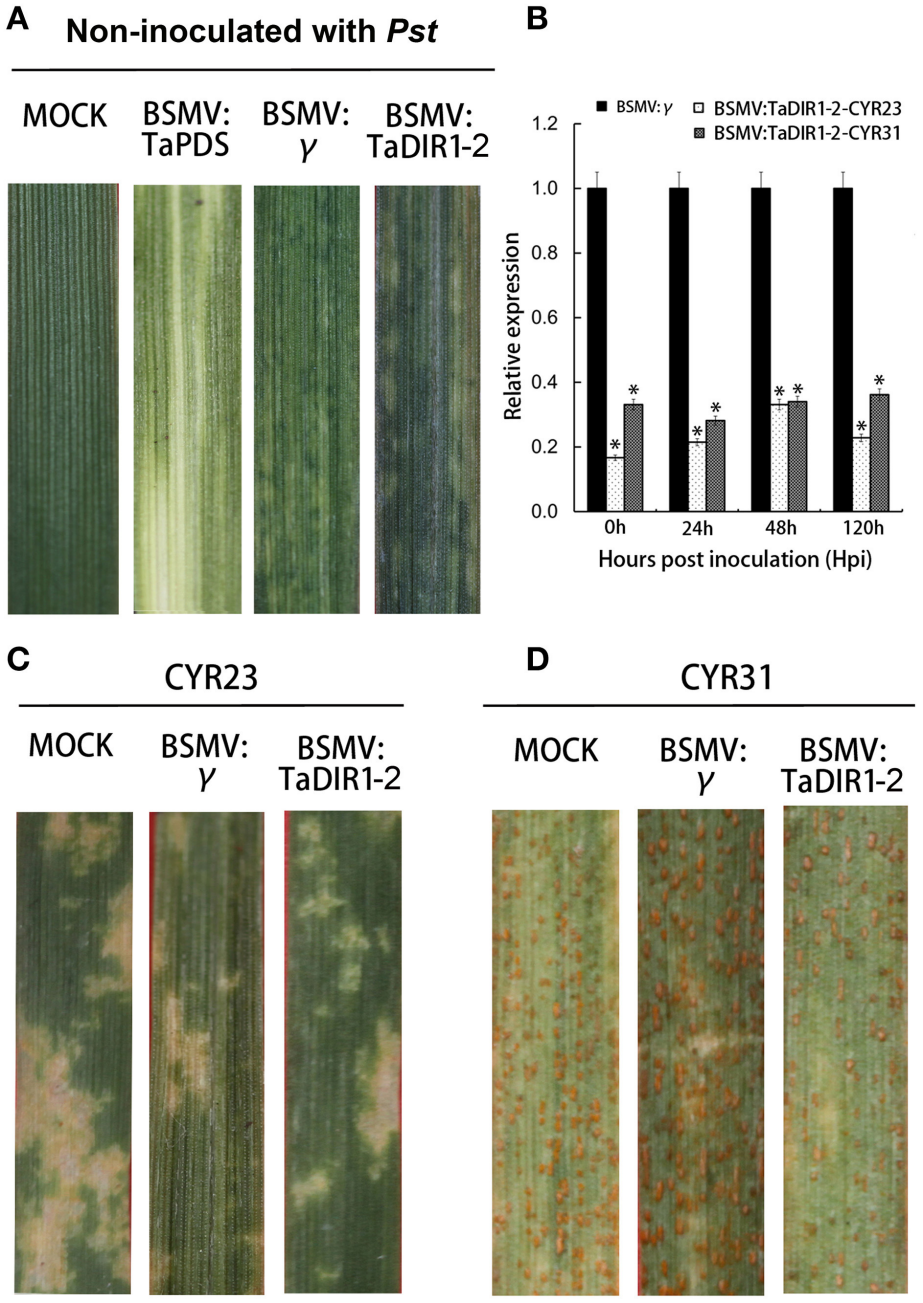
**Functional assessment of TaDIR1-2 in wheat–Pst interaction by the BSMV-VIGS method. (A)** Phenotypes of wheat leaves inoculated with FES buffer as mock; mild chlorotic mosaic symptoms were observed on the fourth leaves of seedlings 10 days post-inoculation(dpi) with BSMV:γ and BSMV:TaDIR1-2and photo bleaching of the fourth leaves of plants infected with BSMV:TaPDSas at 15 dpi. **(B)** Silencing efficiency assessment of TaDIR1-2-knocked-down plants. RNA samples were isolated from the fourth leaves of the wheat seedlings infected by BSMV:γ and BSMV:TaDIR1-2 at 0, 24, 48, and 120 hpi after inoculation with the avirulent (CYR23) or virulent (CYR31) Pst race. Wheat leaves inoculated with BSMV:γ and subsequently challenged by Pst races were used as the controls. Results are shown as means ± standard deviation of three biological replications. Relative gene quantification was calculated by the comparative Δ ΔCT method, and data were normalized to the expression level of the wheat elongation factor TaEF-1α (GenBank accession no. Q03033). Asterisks indicate a significant difference (*P* < 0.05) from the 0 hpi by Student's *t*-test. **(C)** Disease phenotypes of the fourth leaves pre-inoculated with BSMV:γ and then challenged with avirulent CYR23 or **(D)** virulent CYR31 Pst races. Photos were taken 15 days after pathogen inoculation.

The function of *TaDIR1-2* in the wheat defense response against *Pst* was examined by reducing the expression of *TaDIR1-2* through the BSMV-mediated VIGS system (Scofield et al., 2005). To increase the silencing efficiency on TaDIR1-2, a 155-bp fragment was selected and designed from a region of the TaDIR1-2 coding sequence which was relatively conserved in other 11 wheat DIR1 orthologs identified in this study (Supplementary Figure S2; Supplementary Tables S2, S3). In the following experiments, the second leaves of Su11 seedlings at the two-leaf stage were inoculated with BSMV:TaPDSas, BSMV: TaDIR1-2, and BSMV:γ. A similar number of seedlings were also mock-inoculated with a buffer that did not include BSMV. The photo-bleaching phenotype was observed when *TaPDS* (wheat phytoene desaturase gene) was silenced, which provides a visual indication of the occurrence of gene silencing in the leaves after virus inoculation. All the BSMV: TaDIR1-2 and BSMV:γ inoculated plants displayed mild chlorotic mosaic symptoms 10 dpi which did not appear to affect further leaf growth, confirming that the BSMV-mediated gene silencing system functioned correctly and could be used in further experiments. In contrast, the leaves in the mock-inoculated wheat plants developed normally during the observation (Figure 6A). To clarify whether *TaDIR1-2* was successfully silenced, transcript levels of *TaDIR1-2* were examined by qRT-PCR. The results demonstrated that the expression levels of *TaDIR1-2* were decreased at 0, 24, 48, and 120 hpi by approximately 80, 85, 70, and 85% for the incompatible interaction and by 70, 75, 70, and 70% for the compatible interaction, respectively (Figure 6B), indicating that *TaDIR1-2* was successfully silenced.

The fourth leaves in wheat were then inoculated with CYR23 (incompatible) or CYR31 (incompatible). In the incompatible interaction, obvious HR was expressed on mock-inoculated plants and on leaves that were previously infected with BSMV:γ or BSMV:TaDIR1-2 (Figure 6C). In contrast, in the compatible interaction, all leaves from the mock-inoculated plants and leaves that were previously infected with BSMV:γ, revealed a fully susceptible phenotype. Leaves of the TaDIR1-2-knockdown plants also exhibited a susceptible phenotype, but clear necrotic cell death was observed accompanied by reduced sporulation at 15 dpi (Figure 6D). After 15 dpi, 40 leaves infected by Pst were examined for each treatment and they were scored as 10 out of 15 leaves which showed reduced disease symptom. McNeal measurements scale were used to estimate the difference between the phenotypes of BSMV: γ and BSMV:TaDIR1-2 under the interaction with Pst (McNeal et al., 1971) Our results indicated that knockdown of TaDIR1-2 expression increased the resistance of wheat against Pst.

### *Pst* growth and host response

**Figure 7:**
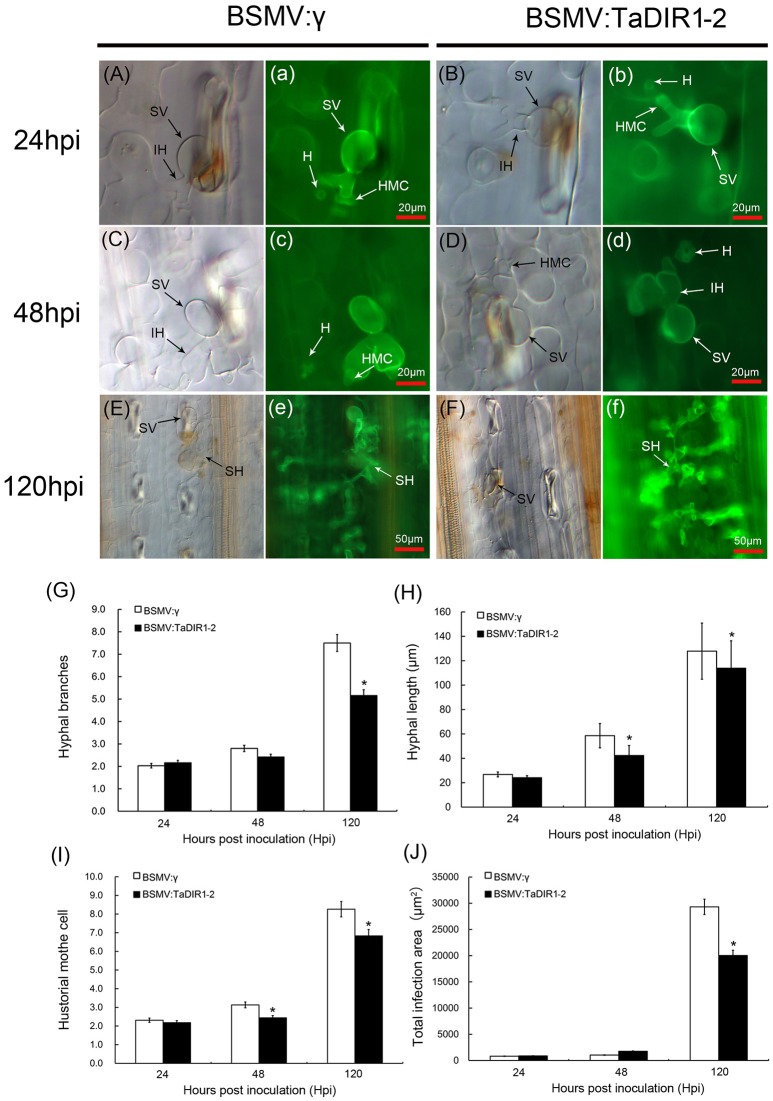
**Histological observation of fungal growth and necrotic cell area in wheat infected with BSMV:γ or BSMV:TaDIR1-2 after inoculation with the Pst virulent pathotype CYR31**. The fungal growth and necrotic cell area in wheat leaves inoculated with BSMV:γ or BSMV:TaDIR1-2at 24 hpi **(Aa,Bb)**, at 48 hpi **(Cc,Dd)**, and at120 hpi **(Ee,Ff)** were observed under a light microscope. The same letter indicates the photo was taken at the same infection site. The average number of hyphal branch and haustorial mother cell **(G,I)** of fungus in each infection site was counted. **(H)** Hyphal length, (the average distance from the junction of the substomatal vesicle and the hypha to the tip of the hypha), was measured using DP-BSW software (unit in μm). **(J)** Infection area (the average area of the expanding hypha) was calculated using DP-BSW software (units of 103 μm2). All results were obtained from 50 infection sites. Asterisks indicate a significant difference (*P* < 0.05) from the BSMV:γ by Student's *t*-test. HMC, haustorial mother cell; HR, hypersensitive reaction; IH, infection hypha; SH, secondary hypha; SV, substomatal vesicle; HMC, hostorial mother cell; H, haustorium.

To determine whether the phenotypic changes in wheat leaves are associated with fungal growth and development, when TaDIR1-2 was knocked down, leaf segments inoculated with CYR23 and CYR31 were examined microscopically (Figures 7A–Ff, Supplementary Figures S4A–Ff). In the compatible interaction, hyphal branches, hyphal length, number of haustorial mother cells, and infected area were not significantly (*P* < 0.05) affected at 24 hpi (Figures 7G–J). However, silencing of TaDIR1-2 significantly decreased hyphal branches, hyphal length, number of haustorial mother cells, and infected area by 120 hpi (Figures 7G–J), respectively, at 48 hpi, the hyphal length and number of haustorial mother cells of Pst were significantly reduced in TaDIR1-2-knockdown plants compared to BSMV:γ infected plants (Figures 7H,I), indicating that knocking down the TaDIR1-2 transcription in wheat plants reduces susceptibility in response to virulent CYR31 infection. In the incompatible interaction, compared with BSMV:γ infected leaves, the number of hyphal branches, hyphal length and number of haustorial mother cells in BSMV:TaDIR1-2 infected leaves were significantly decreased at 24 and 48 hpi, respectively (Supplementary Figures S4G–I). In addition, the infection area in TaDIR1-2-knockdown plants was significantly reduced only at 120 hpi (Supplementary Figure S4J). These histological results indicated that suppression of TaDIR1-2 expression restricts fungal growth and thus enhances host resistance to avirulent and virulent *Pst*.

### Increased accumulation of ROS and SA in *TaDIR1-2*-knockdown plants

**Figure 8:**
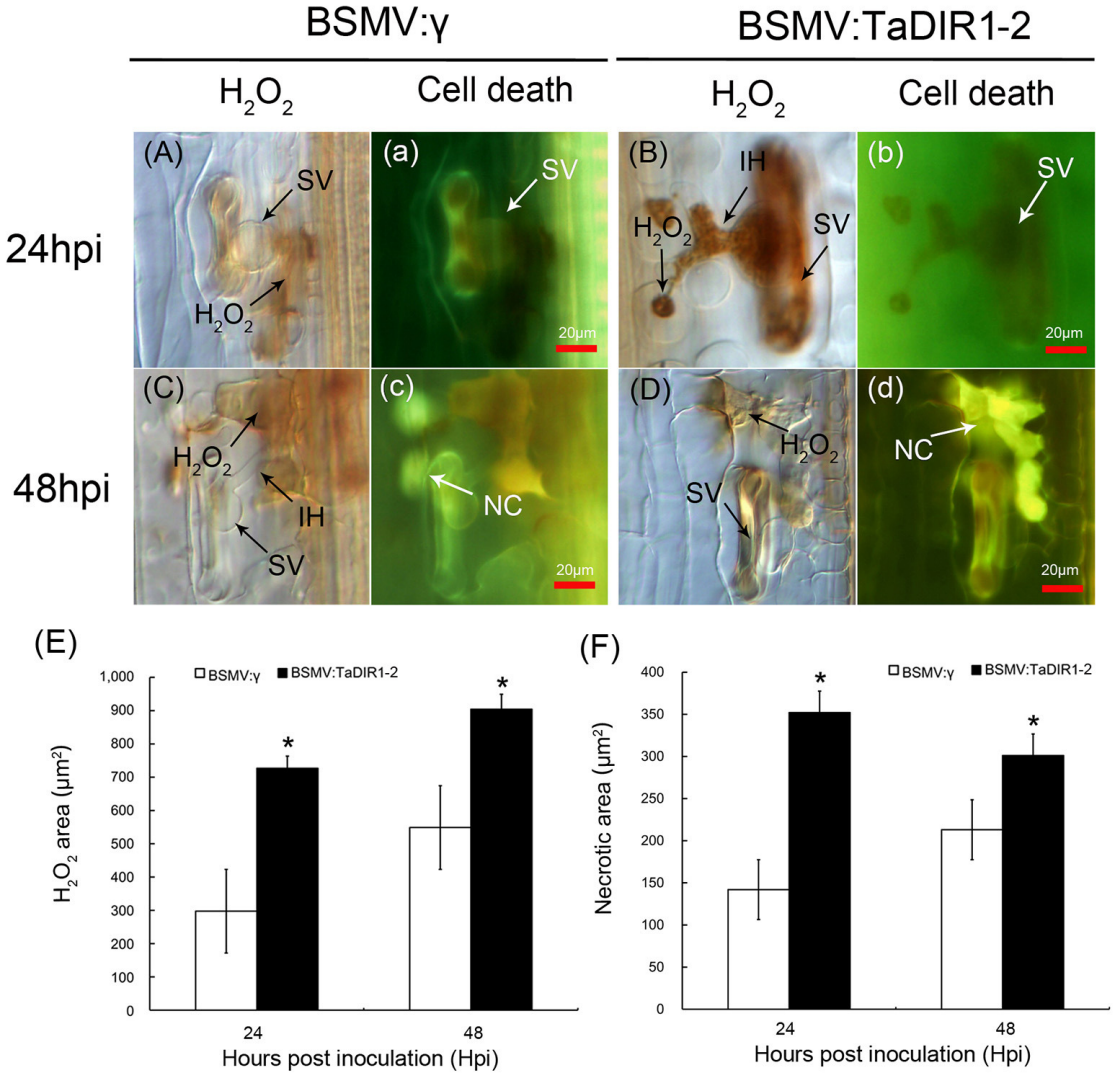
**Increased H_**2**_O_**2**_ accumulation when TaDIR1-2 was silenced and challenged by Pst virulent race CYR31**. H_2_O_2_ accumulation and necrotic cell death were measured in wheat leaves inoculated with BSMV:γ or BSMV:TaDIR1-2at 24 hpi **(Aa,Bb)** and 48 hpi **(Cc,Dd)**. The same letter indicates the photo was taken at the same infection site. For H_2_O_2_ detection, inoculated leaves were stained with 3,3′-diaminobenzidine (DAB) and observed with a light microscope. The necrotic cell death at the same infection site (small letter) was observed under a fluorescence microscope (excitation filter 485 nm, dichromic mirror 510 nm, barrier filter 520 nm). The average area of H_2_O_2_ **(E)** and the necrotic cell death **(F)** were calculated using DP-BSW software (units of 103 μm2). All results were obtained from 50 infection sites. Asterisks indicate a significant difference (*P* < 0.05) from the BSMV:γ by Student's *t*-test. SV, substomatal vesicle; NC, necrotic cell death; IH, infection hypha.

An oxidative burst and the accumulation of ROS with the occurrence of HR are important for resistance against rust fungi in wheat (Dmochowska Boguta et al., 2013). To further clarify the host resistance response in TaDIR1-2-knockdown plants, the induction of H_2_O_2_ during Pst infection was analyzed by histochemical observations after DAB staining. In the compatible interaction, H_2_O_2_ accumulated in TaDIR1-2-knockdown plants to levels about 2.5- and 1.5-fold higher than in the control plants at 24 hpi and 48 hpi (Figures 8A–E), respectively. Similarly, in TaDIR1-2-knockdown plants, the necrotic cell death area observed by auto-fluorescence also exhibited a significantly increased pattern at 24 hpi and 48 hpi (Figures 8a–d,F), respectively. In the incompatible interaction, compared with the control plants, the accumulation of H_2_O_2_ and necrotic cell death areas per infection site were significantly increased both at 24 hpi (Supplementary Figures S5Aa,Bb,E) and 48 hpi (Supplementary Figures S5Cc,Dd,F), respectively.

**Figure 9:**
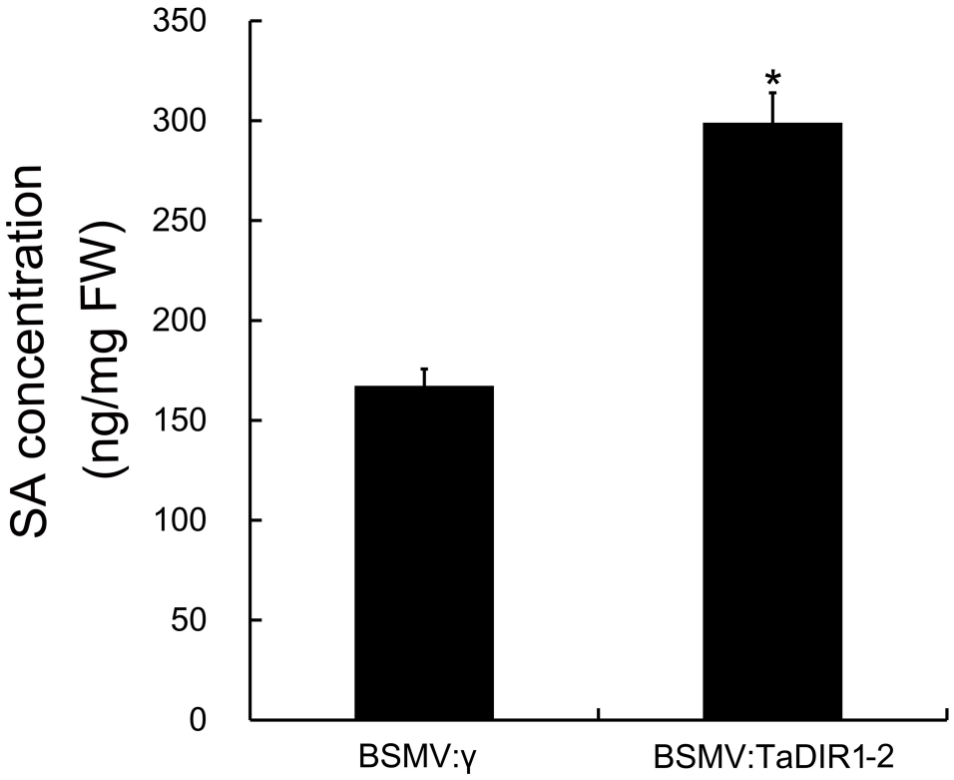
**Quantification of SA level in TaDIR1-2-knock-down plants**. Samples were isolated from the fourth leaves of the wheat seedlings infected by BSMV:γ and BSMV:TaDIR1-2. Results are shown as means ± standard deviation of three biological replications. Asterisks indicate a significant difference (*P* < 0.05) from the BSMV:γ using Student's *t*-test.

To determine whether knockdown of TaDIR1-2 expression can affect SA balance in wheat and thereby lead to increased resistance to Pst, we further quantified the SA content in TaDIR1-2-knockdown plants. As shown in Figure 9, the SA concentration in TaDIR1-2-knockdown plants was significantly increased and reached 300 ng/mg FW, which is 2-fold higher than that in the control plants. Our results suggested that silencing of TaDIR1-2 enhances wheat resistance against Pst by regulating ROS and the SA signaling pathway.

### Transcription of *PR* and ROS-related genes are affected in *TaDIR1-2-*knockdown plants

**Figure 10:**
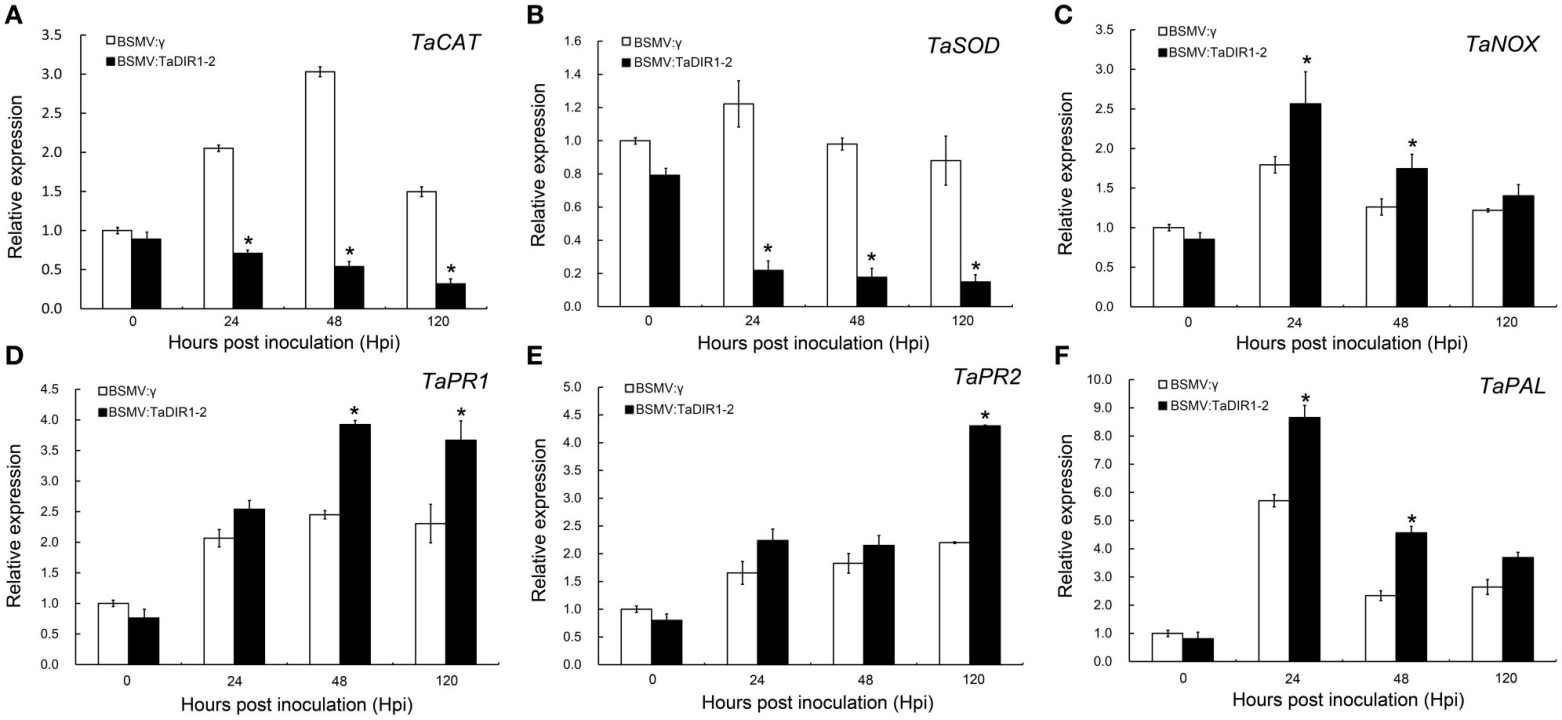
**Transcript profiles of pathogenesis-related (PR) genes and reactive oxygen species (ROS)-related genes in TaDIR1-2-knock-down plants challenged by Pst virulent race CYR31**. Reduced relative expression of ROS scavenging genes catalase (TaCAT) and superoxide dismutase (TaSOD) **(A,B)**, increased expression of ROS-generating gene NADH-oxidase (TaNOX) **(C)**, and increased relative expression of TaPR1, TaPR2 and TaPAL **(D–F)** in TaDIR1-2-knock-down plants. Samples were taken at 0, 24, 48, and 120 hpi from the leaves infected with BSMV:γ or BSMV:TaDIR1-2 after inoculation with the virulent (CYR31) Pst race. Transcript levels of BSMV:γ at 0 hpi were set to one. Results are shown as means ± standard deviation of three biological replications. Relative gene quantification was calculated by the comparative ΔΔCt method. Data were normalized to the expression level of the wheat elongation factor TaEF-1α (GenBank accession no. Q03033). Asterisks indicate a significant difference (*P* < 0.05) from the BSMV:γ using Student's *t*-test.

To confirm the increased resistance and ROS accumulation in TaDIR1-2-knockdown plants, the expression of three ROS-related genes, two PR protein genes (PR1 and PR2), as marker genes for SA as well as in HR (Van Loon, 1997) and a defense-related secondary metabolite gene phenylalanine ammonia-lyase (PAL) were examined by qRT-PCR (Figure 10, Supplementary Figure S6). In compatible and incompatible interactions, the transcript levels of ROS-scavenging genes, both TaCAT (Figure 10A, Supplementary Figure S6A) and TaSOD (Figure 10B, Supplementary Figure S6B), were significantly decreased at 24, 48 and 120 hpi in TaDIR1-2-knockdown plants compared with the controls plants. TaNOX, a ROS-generating gene, exhibited a significantly increased expression at 24 and 48 hpi in TaDIR1-2-silenced plants compared to the control plants in compatible (Figure 10C) and incompatible (Supplementary Figure S6C) interactions. In addition, in compatible interaction the transcription of TaPR1 and TaPR2, were significantly induced at 48 and 120 hpi in TaDIR1-2 knockdown plants, respectively (Figures 10D,E), and in incompatible interactions significant induction were showed at 24 hpi (Supplementary Figures S6D,E). The transcript levels of the TaPAL were dramatically induced as early as 24 hpi in TaDIR1-2-knockdown plants in both the compatible (Figure 10F) and incompatible (Supplementary Figure S6F) interactions. In addition, at 0 hpi, which was considered as without a pathogen attack stage, no change in transcript levels of ROS related genes, PR protein genes or PAL gene were observed in TaDIR1-2-knockdown plants compared with the control plants (Figure 10 and Supplementary Figure S6), indicating that viral infection was not involved in defense response and the knockdown of TaDIR1-2 expression resulted in H_2_O_2_ as well as SA accumulation during Pst infection. These results further suggested that knockdown of TaDIR1-2 expression enhances the resistance of wheat to the stripe rust fungus in ROS- and SA-dependent manner.

## Discussion

Many studies have shown the importance of AtDIR1 from Arabidopsis and its orthologs from dicot plants in the SAR response (Maldonado et al., 2002; Isaacs et al., 2016). Heterologous expression of AtDIR1, OsDIR1-A, and OsDIR1-B in barley appeared to affect local defense gene expression and symptom development, which suggests that DIR1 is an excellent candidate for investigation on its role in acquired resistance in the cereals (Colebrook, 2010). In this study, we firstly characterized a AtDIR1 ortholog in wheat, designated TaDIR1-2, and further suggests that it performs a negative role in wheat immunity during the wheat-Pst interaction.

Polyploidy is a prominent speciation process in plants that results in genome-wide gene duplication (Wendel, 2000). Wheat, which is mostly hexaploid with three sub-genomes (A, B, and D), is a typical example of allopolyploidization (Wendel, 2000). In the present study, we identified 5, 8, and 2 duplicated repeat segments in the putative DIR1 protein sequences from wheat in the A, B, and D sub-genomes, respectively. This result suggests the existence of tandem duplication event of the DIR1 gene in wheat. Accordingly, Champigny et al. (2013) suggested that AtDIR1 and AtDIR1-like arose from a tandem duplication event. In addition, independent DIR1 duplications have been documented in other dicot plants, such as tomato, cucumber, tobacco, and soybean (Isaacs et al., 2016). Our study indicated that putative DIR1 orthologs identified from wheat were clustered into six major groups. Phylogenetic analysis revealed that TaDIR1-2 in the DIR1-2 group has a more distant relationship with AtDIR1 than DIR1-1 orthologs, suggesting that it is paralogous to AtDIR1 and preserves its unique function.

Differential transcriptions of LTPs have been shown in a variety of plants and plant tissues (de Oliveira Carvalho and Gomes, 2007). Six TaLTP genes were observed to be highly active in leaves of transgenic rice (Boutrot et al., 2007). In Arabidopsis, LTP1 was expressed in epidermal cells of young leaves and the stem (Thoma et al., 1994). In addition, DIR1 is expressed constitutively to low levels in all the tissues tested including seedlings, roots and flowers and in the living cells in leaves of Arabidopsis (Champigny et al., 2011). Consistent with that observation, the transcription of TaDIR1-2 was also detected in all vegetative organs tested, suggesting that its expression is more likely ubiquitous in wheat plants. The LTPs are often reported to be important in response to abiotic stresses, such as drought, cold, and salt or to wounding in a ROS-activated defense pattern (Gangadhar et al., 2016; Salminen et al., 2016). Significantly increased expression of TaDIR1-2 under low temperature (4°C) treatment suggests the involvement of TaDIR1-2 in enhancing plant acclimation to abiotic stresses. Cold-inducible expression of LTPs in wheat (Yu et al., 2014), rice (Guo C. et al., 2013), maize (Wei and Zhong, 2014), and Arabidopsis (Xu et al., 2011), indicated that LTPs are strongly associated with cold tolerance in higher plants. It is possible that TaDIR1-2 contributes to cold tolerance by interacting with other proteins to modulate defense responses to abiotic stresses in a ROS-dependent manner.

The HR, a common feature of gene-for-gene resistance in plants to various pathogens, has also been described as a form of PCD, and ROS have been considered as key factors of the induction and modulation of the PCD during plant-pathogen interaction (Richberg et al., 1998; Gadjev et al., 2008). During the induction of basal resistance or SAR, DIR1/LTPs are proposed to form a complex between a target protein and a lipid molecule which are released on the action of secreted lipases following a pathogen attack (Champigny and Cameron, 2009). As such, in Arabidopsis, overexpression of DIR1 or MYB30 could not induce basal resistance or spontaneous HR-like lesions (Maldonado et al., 2002; Vailleau et al., 2002). When TaDIR1-2 was overexpressed in N. benthamiana cells, TaDIR1-2 also could not induce PCD in tobacco leaves, perhaps due to the absence of pathogen infection. Bax-induced cell death is the result of mitochondrial lipid oxidation, which may be prevented by the co-expression of lipid oxidation inhibitors (Priault et al., 2002). Thus, it may be assumed that TaDIR1-2 lacks a mitochondrial lipid oxidation inhibition function to suppress BAX-induced PCD.

Virus-induced gene silencing is a powerful reverse genetics tool that exploits an RNA-mediated antiviral defense mechanism, circumvents the need for plant transformation, is methodologically simple and yields rapid results for high-throughput functional genomics (Lu et al., 2003). In this study, we used a VIGS approach to determine the role of TaDIR1-2 in the wheat-Pst interaction. In response to the virulent Pst CYR31, TaDIR1-2-knockdown plants were less susceptible with increased necrotic area per infection site and decreased hyphal length and sporulation, indicating that silencing of TaDIR1-2 enhanced wheat resistance to virulent Pst. Consistent with that observation, qRT-PCR assays also revealed that the expression of TaDIR1-2 was induced during the compatible interaction, whereas there was no significant change in the incompatible interaction, further suggesting TaDIR1-2 acts as a negative regulator of defense in wheat against Pst. The necrotic cell death area induced by infection of Pst was used to represent the level of the HR, which is probably triggered by generation of ROS during the initial (12–24 hpi) and late (96–120 hpi) infection stages in the wheat-Pst pathosystem (Wang et al., 2007; Zurbriggen et al., 2010). Silencing of TaDIR1-2 expression resulted in a significantly increased accumulation of H_2_O_2_ and/or necrotic cell death in the compatible and incompatible interactions, indicating that suppression of the TaDIR1-2 expression is able to increase cell death, limit pathogen spread, and thereby enhance disease resistance to Pst infection via regulating the ROS-dependent signaling pathway. ROS signaling in cells is a result of the contrasting processes of ROS generation and scavenging in a simultaneous manner (Mittler et al., 2011). Transcription levels of TaSOD and TaCAT were significantly reduced in Pst-infected TaDIR1-2-knockdown plants, while the ROS-generating gene TaNOX was highly induced, further supporting the notion that the function of TaDIR1-2 in wheat defense against Pst is ROS-dependent.

In combating microbial pathogens, plants employ two approaches of their innate immune system, PTI and ETI, which appeared to be mediated by an integrated signaling network (Tsuda and Katagiri, 2010). SA is a signaling molecule involved in basal immunity against biotrophic pathogen infection (Glazebrook, 2005). In addition, PAL a defense related gene and the PR proteins are proposed as marker genes for HR and SA signal pathway (Mauch-Mani and Slusarenko, 1996; Van Loon, 1997). Our results indicated that a significant SA accumlation and up-regulation of TaPR1, TaPR2, and TaPAL upon Pst infection in TaDIR1-2-silenced plants, suggesting that silencing of TaDIR1-2 expression leads to increased resistance to Pst by regulating the SA signaling pathway in wheat.

Considerable evidence has documented that DIR1 is a cell wall protein and promotes long distance signaling during SAR (Champigny et al., 2011). However, heterologous expression of AtDIR1, OsDIR1-A, or OsDIR1-B in barley could not induce acquired resistance (Colebrook, 2010). In this study, our data also suggest that TaDIR1-2 acts as a negative regulator in local resistance of wheat to Pst. Therefore, more work must be performed to establish whether TaDIR1-2 has a role during SAR in wheat.

To our knowledge, this is the first report to suggest that DIR1 orthologs negatively regulate the resistance against pathogen infection in plants. On the basis of our experimental results, we propose that TaDIR1-2 likely acts as a negative regulator in wheat resistance to Pst by modulating ROS and/or SA-induced signaling. In Arabidopsis LTP3 has been proved to be a novel negative regulator of plant immunity which acts through the manipulation of the ABA-SA balance and supposed to be targeted by Pseudomonas effector(s) in order to disturb the ABA pathway (Gao et al., 2015). Further identification of the upstream or downstream interacting targets of TaDIR1-2 to regulate the ROS- or SA-mediated cell death may provide valuable insight into the molecular mechanism of plant immunity.

## Author contributions

JuG and ZK designed the experiment. SA, PL, QX, CJ, TQ, and JiG performed the experiments and analyzed the data. SA, PL, JuG, and ZK wrote the manuscript.

## Funding

This study was financially supported by National Basic Research Program of China (No.2013CB127700), National Natural Science Foundation of China (No.31371889 and 31171795), the Program for New Century Excellent Talents in University (NCET-12-0471), the 111 Project from the Ministry of Education of China (No.B07049), the Natural Science Foundation of Shaanxi Province (2014JM3059) and the Fundamental Research Funds for the Central Universities of China (YQ2013001).

### Conflict of interest statement

The authors declare that the research was conducted in the absence of any commercial or financial relationships that could be construed as a potential conflict of interest.

## Supplementary material

Supplementary Figure S1 **A representative phylogenetic tree of TaDIR1-2 with DIR1 wheat genomic sequences and other higher plants**. Branches are labeled with the protein names, ID number and GenBank accession numbers. At, *Arabidopsis thaliana*; EMT, ID number of Aegilops tauschii genome database; Os, *Oryza sativa*; Ta, *Triticum aestivum*; TRIAE, ID number of Chinese Spring wheat genome database; TRIUR, ID number of *Triticum urartu*genome database. Bootstrap values (>50%) based on 1,000 replications are shown at branch nodes. The bar indicates 1 substitution per 10 nucleotide positions.

Supplementary Figure S2 **Multiple sequence alignment of TaDIR1-2 with other wheat DIR1 orthologs**. Alignments of **(A)** DNA sequences and **(B)** amino acid sequences of TaDIR1-2 with other wheat DIR1 orthologs. Red underline: fragment (155 bp) of TaDIR1-2 for VIGS analysis; red box and blue box indicating the initiation codon (ATG) and termination codon (TGA), respectively. Sequence was obtained from the International Wheat Genome Sequencing Consortium database and alignment was performed using DNAMAN software.

Supplementary Figure S3 **Transient overexpression of eGFP in tobacco leaf**. Tobacco leaf cell from the area infiltrated with PVX-eGFP was examined with a laser scanning microscope.

Supplementary Figure S4 **Histological observations of fungal growth and necrotic cell area in wheat infected with BSMV:γ or BSMV:TaDIR1-2 after inoculation with the Pst avirulent pathotype CYR23**. The fungal growth and necrotic cell area in wheat leaves inoculated with BSMV:γ or BSMV:TaDIR1-2 at 24 hpi **(Aa,Bb)**, at 48 hpi **(Cc, Dd)**, and at 120 hpi **(Ee, Ff)** were observed under a light microscope. The same letter indicates the photo was taken at the same infection site. The average number of hyphal branches and haustorial mother cells **(G,I)** of the pathogenin each infection site was determined. **(H)** Hyphal length, (the average distance from the junction of the substomatal vesicle and the hypha to the tip of the hypha), was measured using DP-BSW software (units in μm). **(J)** Infection area (the average area of the expanding hypha) was calculated using DP-BSW software (units of 103 μm2). All results were obtained from 50 infection sites. Asterisks indicate a significant difference (*P* < 0.05) from the BSMV:γ by Student's *t*-test. HMC, haustorial mother cell; HR, hypersensitive reaction; IH, infection hypha; SH, secondary hypha; SV, substomatal vesicle; HMC, haustorial mother cell; H, haustorium.

Supplementary Figure S5 **Increased H_2_O_2_ accumulation when TaDIR1-2 was silenced and challenged by Pst avirulent race CYR23**. H_2_O_2_ accumulation and necrotic cell death were measured in wheat leaves inoculated with BSMV:γ or BSMV:TaDIR1-2 at 24 hpi **(Aa,Bb)** and 48 hpi **(Cc,Dd)**. The same letter indicates photos of the same infection site. For H_2_O_2_ detection, inoculated leaves were stained with 3,3′-diaminobenzidine (DAB) and observed with a light microscope. The necrotic cell death at the same infection site (small letter) was observed under a fluorescence microscope (excitation filter 485 nm, dichromic mirror 510 nm, barrier filter 520 nm). The average areas of H_2_O_2_ **(E)** and the necrotic cell death **(F)** were calculated using DP-BSW software (units of 103 μm2). All results were obtained from 50 infection sites. Asterisks indicate a significant difference (*P* < 0.05) from the BSMV:γ by Student's *t*-test. SV, substomatal vesicle; NC, necrotic cell death.

Supplementary Figure S6 **Transcript profiles of pathogenesis-related protein (PR) genes and reactive oxygen species (ROS)-related genes in TaDIR1-2-knocked-down plants challenged by Pst avirulent race CYR23**. Reduced relative expression of ROS scavenging genes catalase (TaCAT) and superoxide dismutase (TaSOD) **(A,B)**, increased expression of ROS-generating gene NADH-oxidase (TaNOX) **(C)**, and increased relative expression of TaPR1, TaPR2 and TaPAL **(D–F)** in TaDIR1-2-knock-down plants. Samples were taken at 0, 24, 48, and 120 hpi from leaves infected with BSMV:γ or BSMV:TaDIR1-2after inoculation with the avirulent (CYR23) Pst race. Transcript levels of BSMV:γ at 0 hpi were set to one. Results were shown as means ± standard deviation of three biological replications. Relative gene quantification was calculated by the comparative ΔΔCt method. Data were normalized to the expression level of the wheat elongation factor TaEF-1α (GenBank accession no. Q03033). Asterisks indicate a significant difference (*P* < 0.05) from the BSMV:γ using Student's *t*-test.

Supplementary Table S1 **Primers used for PCR and plasmid construction**.

Supplementary Table S2 **Nucleotide (VIGS fragment) identities of *TaDIR1* orthologs sequences to BSMV:TaDIR1-2**.

Supplementary Table S3 **BSMV: TaDIR1-2 silencing targets predicted by si-Fi analysis**.
