# Supplementary figures and images for: TaDIR1-2, a Wheat Ortholog of Lipid Transfer Protein AtDIR1 Contributes to Negative Regulation of Wheat Resistance against Puccinia striiformis f. sp. tritici
Source: Front Plant Sci. 2017 Apr 11;8:521. doi: 10.3389/fpls.2017.00521 (PMC5387106; doi:10.3389/fpls.2017.00521)

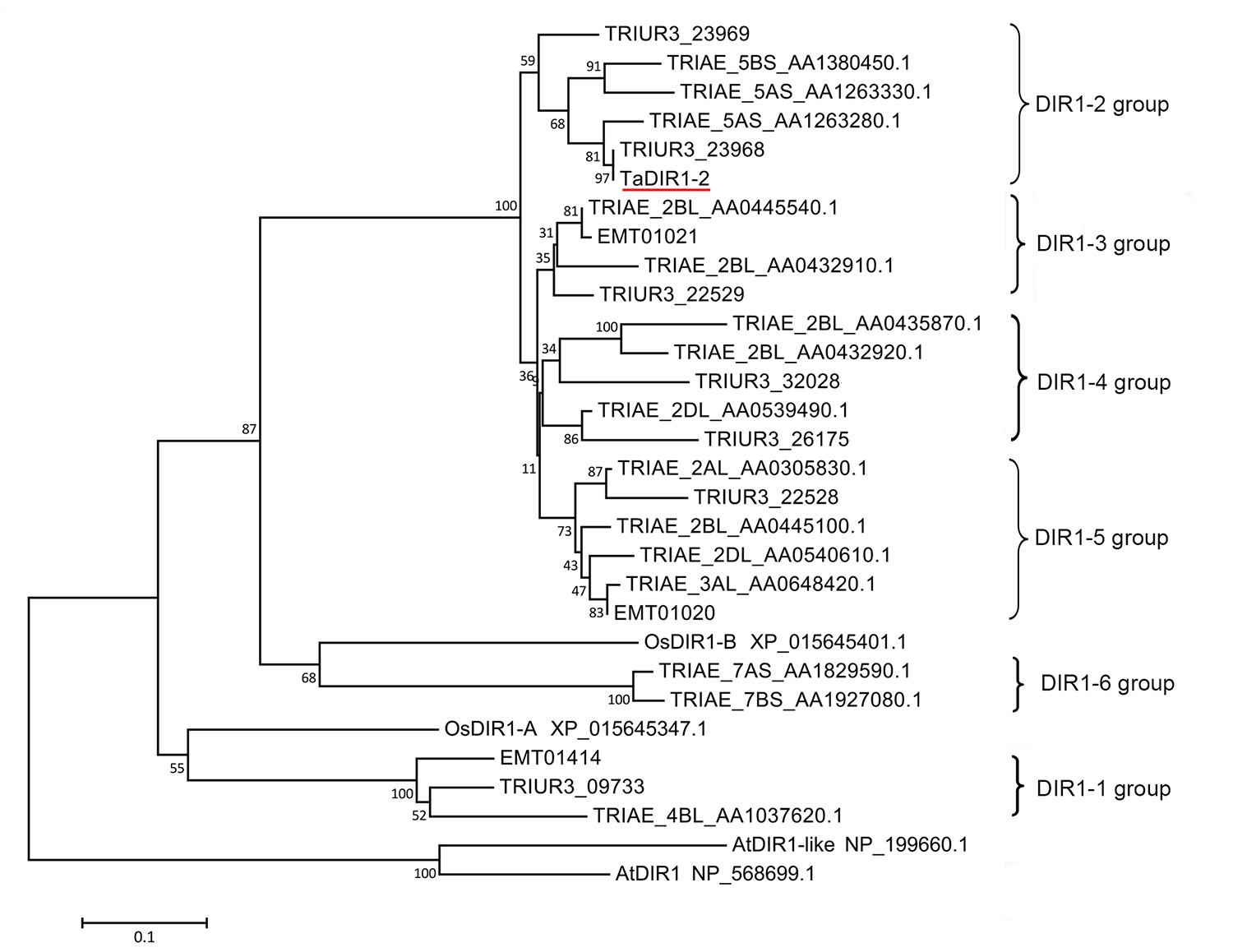

Supplement: Supplementary Figure S1 — A representative phylogenetic tree of TaDIR1-2 with DIR1 wheat genomic sequences and other higher plants. Branches are labeled with the protein names, ID number and GenBank accession numbers. At, Arabidopsis thaliana; EMT, ID number of Aegilops tauschii genome database; Os, Oryza sativa; Ta, Triticum aestivum; TRIAE, ID number of Chinese Spring wheat genome database; TRIUR, ID number of Triticum urartugenome database. Bootstrap values (>50%) based on 1,000 replications are shown at branch nodes. The bar indicates 1 substitution per 10 nucleotide positions. [file Image1.JPEG]

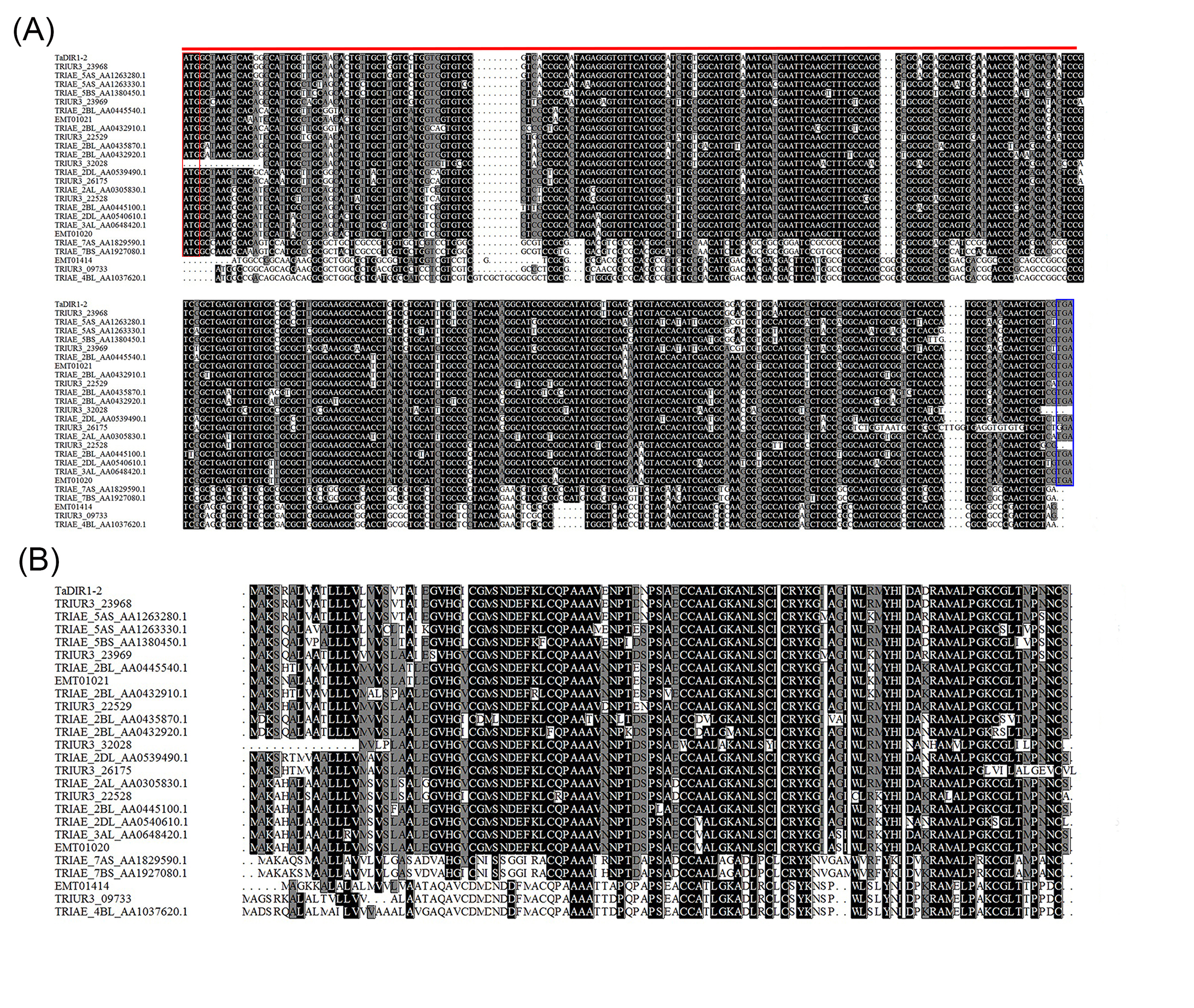

Supplement: Supplementary Figure S2 — Multiple sequence alignment of TaDIR1-2 with other wheat DIR1 orthologs. Alignments of (A) DNA sequences and (B) amino acid sequences of TaDIR1-2 with other wheat DIR1 orthologs. Red underline: fragment (155 bp) of TaDIR1-2 for VIGS analysis; red box and blue box indicating the initiation codon (ATG) and termination codon (TGA), respectively. Sequence was obtained from the International Wheat Genome Sequencing Consortium database and alignment was performed using DNAMAN software. [file Image2.JPEG]

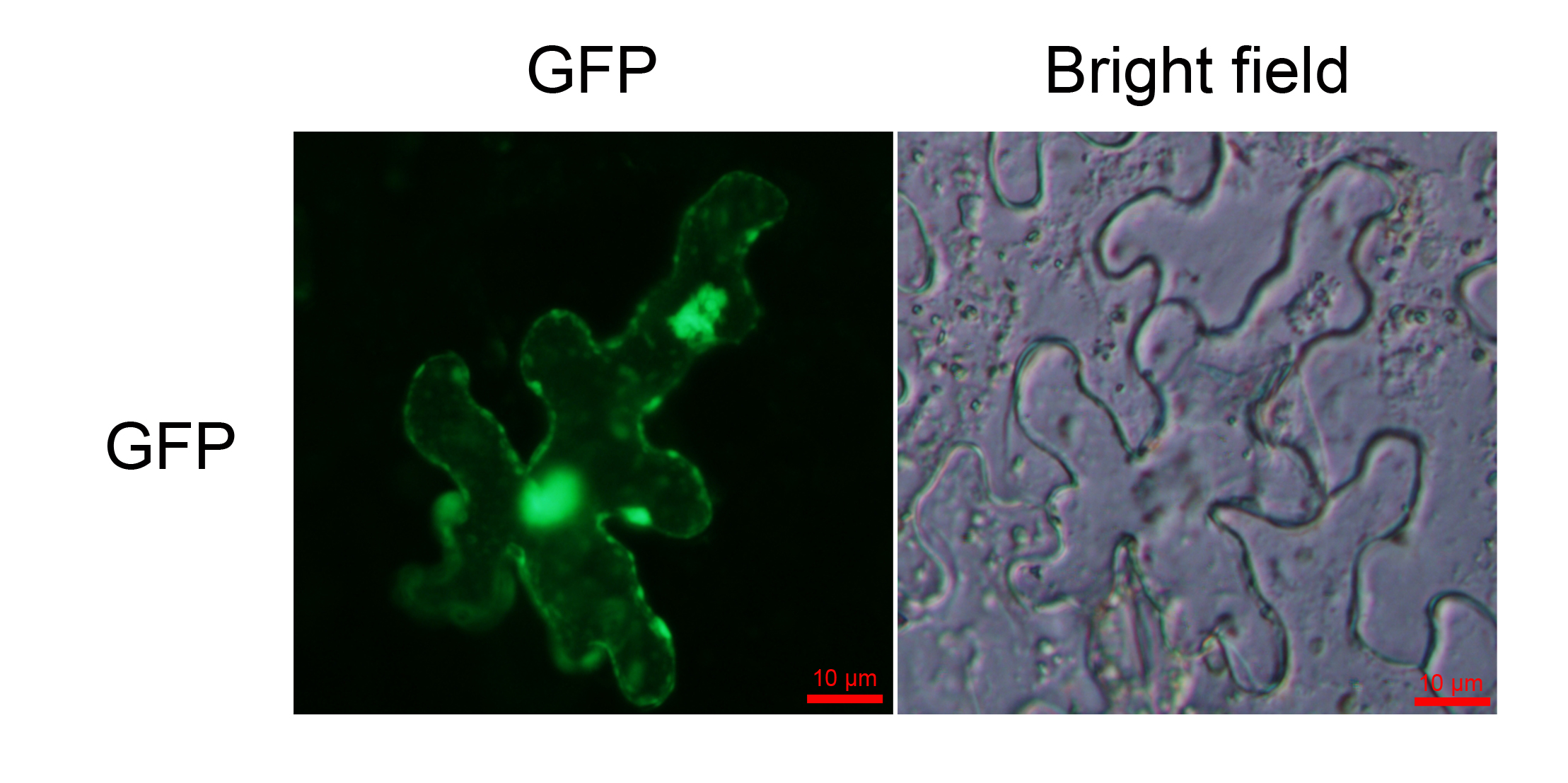

Supplement: Supplementary Figure S3 — Transient overexpression of eGFP in tobacco leaf. Tobacco leaf cell from the area infiltrated with PVX-eGFP was examined with a laser scanning microscope. [file Image3.JPEG]

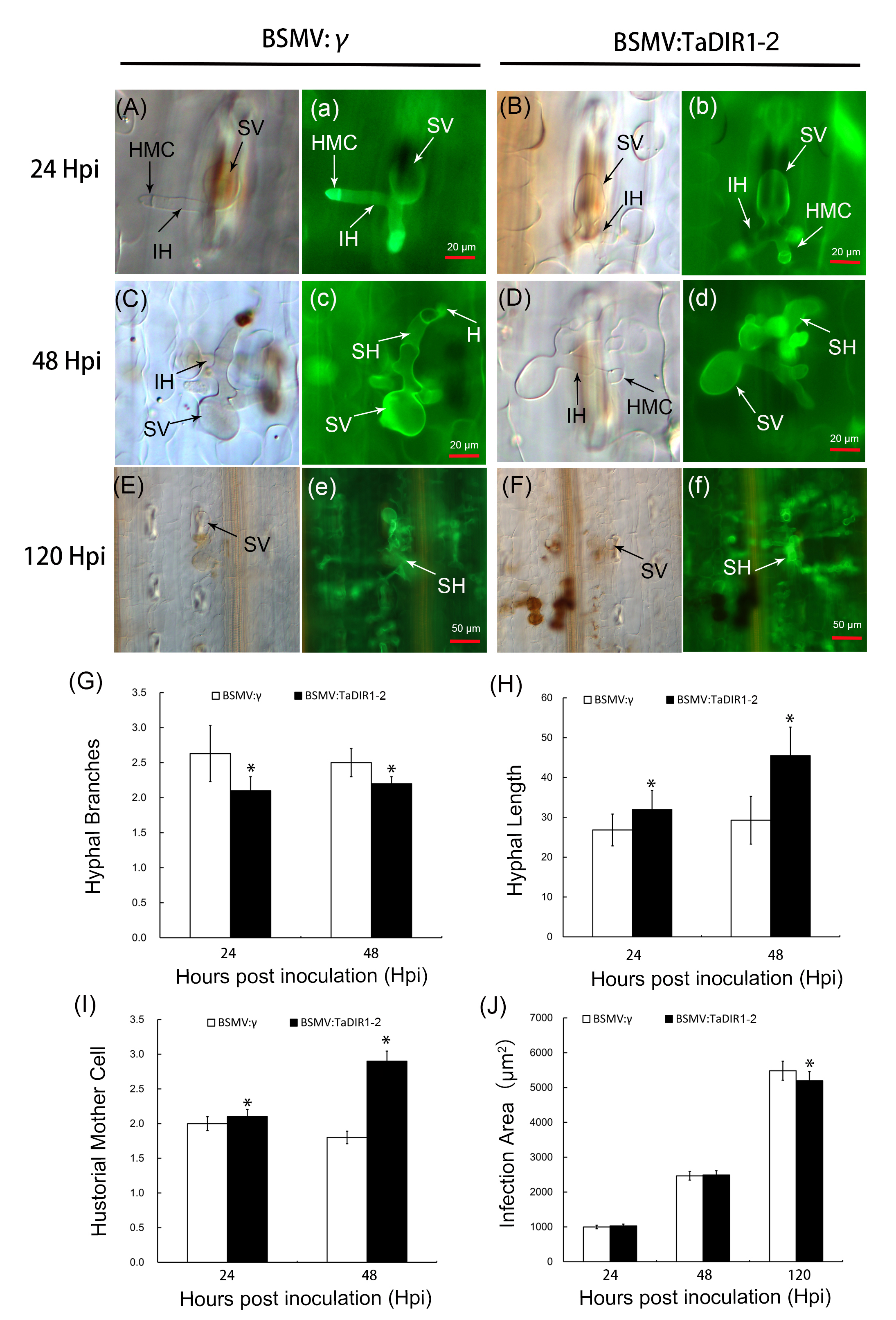

Supplement: Supplementary Figure S4 — Histological observations of fungal growth and necrotic cell area in wheat infected with BSMV:γ or BSMV:TaDIR1-2 after inoculation with the Pst avirulent pathotype CYR23. The fungal growth and necrotic cell area in wheat leaves inoculated with BSMV:γ or BSMV:TaDIR1-2 at 24 hpi (Aa,Bb), at 48 hpi (Cc, Dd), and at 120 hpi (Ee, Ff) were observed under a light microscope. The same letter indicates the photo was taken at the same infection site. The average number of hyphal branches and haustorial mother cells (G,I) of the pathogenin each infection site was determined. (H) Hyphal length, (the average distance from the junction of the substomatal vesicle and the hypha to the tip of the hypha), was measured using DP-BSW software (units in μm). (J) Infection area (the average area of the expanding hypha) was calculated using DP-BSW software (units of 103 μm2). All results were obtained from 50 infection sites. Asterisks indicate a significant difference (P < 0.05) from the BSMV:γ by Student's t-test. HMC, haustorial mother cell; HR, hypersensitive reaction; IH, infection hypha; SH, secondary hypha; SV, substomatal vesicle; HMC, haustorial mother cell; H, haustorium. [file Image4.jpg]

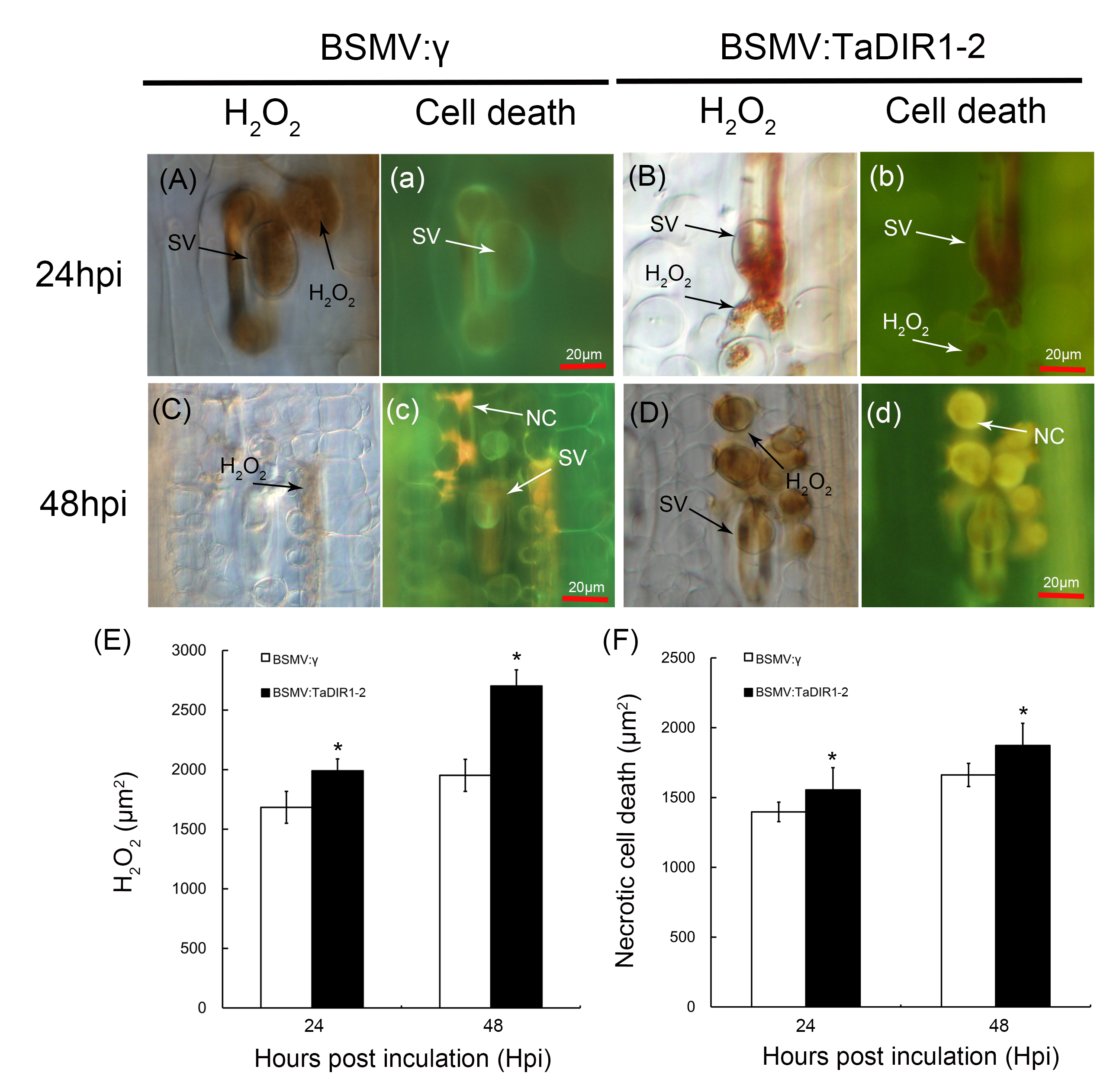

Supplement: Supplementary Figure S5 — Increased H2O2 accumulation when TaDIR1-2 was silenced and challenged by Pst avirulent race CYR23. H2O2 accumulation and necrotic cell death were measured in wheat leaves inoculated with BSMV:γ or BSMV:TaDIR1-2 at 24 hpi (Aa,Bb) and 48 hpi (Cc,Dd). The same letter indicates photos of the same infection site. For H2O2 detection, inoculated leaves were stained with 3,3′-diaminobenzidine (DAB) and observed with a light microscope. The necrotic cell death at the same infection site (small letter) was observed under a fluorescence microscope (excitation filter 485 nm, dichromic mirror 510 nm, barrier filter 520 nm). The average areas of H2O2 (E) and the necrotic cell death (F) were calculated using DP-BSW software (units of 103 μm2). All results were obtained from 50 infection sites. Asterisks indicate a significant difference (P < 0.05) from the BSMV:γ by Student's t-test. SV, substomatal vesicle; NC, necrotic cell death. [file Image5.jpg]

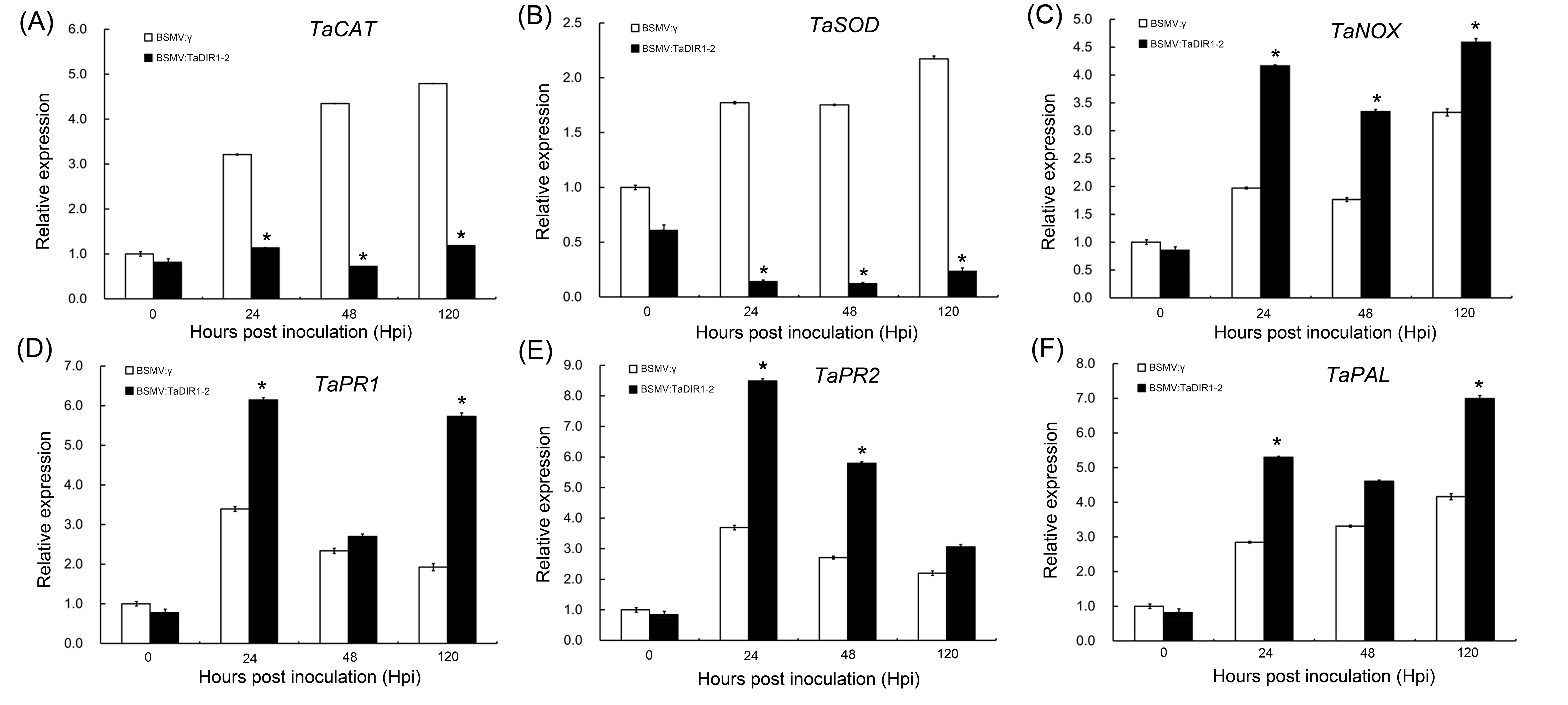

Supplement: Supplementary Figure S6 — Transcript profiles of pathogenesis-related protein (PR) genes and reactive oxygen species (ROS)-related genes in TaDIR1-2-knocked-down plants challenged by Pst avirulent race CYR23. Reduced relative expression of ROS scavenging genes catalase (TaCAT) and superoxide dismutase (TaSOD) (A,B), increased expression of ROS-generating gene NADH-oxidase (TaNOX) (C), and increased relative expression of TaPR1, TaPR2 and TaPAL (D–F) in TaDIR1-2-knock-down plants. Samples were taken at 0, 24, 48, and 120 hpi from leaves infected with BSMV:γ or BSMV:TaDIR1-2after inoculation with the avirulent (CYR23) Pst race. Transcript levels of BSMV:γ at 0 hpi were set to one. Results were shown as means ± standard deviation of three biological replications. Relative gene quantification was calculated by the comparative ΔΔCt method. Data were normalized to the expression level of the wheat elongation factor TaEF-1α (GenBank accession no. Q03033). Asterisks indicate a significant difference (P < 0.05) from the BSMV:γ using Student's t-test. [file Image6.JPEG]
