# Supplementary material for: TaDIR1-2, a Wheat Ortholog of Lipid Transfer Protein AtDIR1 Contributes to Negative Regulation of Wheat Resistance against Puccinia striiformis f. sp. tritici
Source: Front Plant Sci. 2017 Apr 11;8:521. doi: 10.3389/fpls.2017.00521 (PMC5387106; doi:10.3389/fpls.2017.00521)
Supplement: Supplementary Table S1 — Primers used for PCR and plasmid construction. [file Table1.DOC]

**Supplementary Table S1**. Primers used for PCR and plasmid construction.

| **Applications** | **Primers** | **Sequence 5' to 3'** |
| --- | --- | --- |
| cDNA and DNA amplification | TaDIR1-2(ORF)-S | ATGGCTAAGTCACGGGCATTG |
|  | TaDIR1-2(ORF)-AS | TCACGAGCAGTTGTTGGGCAT |
| qRT-PCR | TaDIR1-2(qRT)-S | GGCTAAGTCACGGGCATTG |
|  | TaDIR1-2(qRT)-AS | ACGGATTGTCTGTTGGGTTTT |
|  | TaDIR1-2 (VIGSqRT)-S | CTGTCGTGCATTTGTCGC |
|  | TaDIR1-2(VIGSqRT)-AS | AGTTGTTGGGCATGGTGAG |
|  | TaPR1-S | GAGAATGCAGACGCCCAAGC |
|  | TaPR1-AS | CTGGAGCTTGCAGTCGTTGATC |
|  | TaPR2-S | AGGATGTTGCTTCCATGTTTGCCG |
|  | TaPR2-AS | AAGTAGATGCGCATGCCGTTGATG |
|  | TaPAL-S | CGTCAAGAGCTGTGTGAAGATGG |
|  | TaPAL-AS | GGTAGTTGGAGCTGCAAGGGTC |
|  | TaCAT-S | TGCCTGTGTTTTTTATCCGAGA |
|  | TaCAT-AS | CTGCTGATTAAGGTGTAGGTGTTGA |
|  | TaSOD-S | CCGAGGTCTGGAACCATCAC |
|  | TaSOD-AS | AGCCGAAATCCTTCTCGATCT |
|  | TaNOX-S | ATGTTCGGCAACTTGGTGACT |
|  | TaNOX-AS | CGTCTGCTCTAAGAAGACCACTTTT |
|  | TaEF-1a-F | TGGTGTCATCAAGCCTGGTATGGT |
|  | TaEF-1a-R | ACTCATGGTGCATCTCAACGGACT |
| Subcellular Localization | TaDIR1-2(163)-S | CTGCAGATGGCTAAGTCACGGGCA |
|  | TaDIR1-2(163)-AS | TCTAGACGAGCAGTTGTTGGGCAT |
| VIGS | TaDIR1-2(VIGS)-S | ATATTAATTAAGGCTAAGTCACGGGCATTG |
|  | TaDIR1-2(VIGS)-AS | TATGCGGCCGCACGGATTGTCTGTTGGGTTTT |
| Overexpression in Tobacco | TaDIR1-2(PVX)-S | CCATCGAT ATGGCTAAGTCACGGGCATTGG |
|  | TaDIR1-2(PVX)-AS | ACGCGTCGAC TCACGAGCAGTTGTTGGGCAT |
|  | Avr1b(PVX)-S | CCATCGAT TCACGAACCATGCGTCTATC |
|  | Avr1b(PVX)-AS | ACGCGTCGAC CCCTCGAGATTTCGGCGAGACCTTCTCA |
|  | eGFP(PVX)-S | CCATCGAT ATGGTGAGCAAGGGCGAG |
|  | eGFP(PVX)-AS | ACGCGTCGAC TTACTTGTACAGCTCGTCCATGC |
|  | Bax(PVX)-S | CCATCGAT ATGGACGGGTCCGG |
|  | Bax(PVX)-AS | ACGCGTCGAC GCCCATCTTCTTCCAGAT |
|  | pBIN^19^(PVX)-S | CCATCGAT CGAACAAGTCAATAAACCATGG |
|  | pBIN^19^(PVX)-AS | ACGCGTCGACCCGGAGCTCAGAGTCTGTCTTACTCGCCTTCT |
