# Supplementary material for: TaDIR1-2, a Wheat Ortholog of Lipid Transfer Protein AtDIR1 Contributes to Negative Regulation of Wheat Resistance against Puccinia striiformis f. sp. tritici
Source: Front Plant Sci. 2017 Apr 11;8:521. doi: 10.3389/fpls.2017.00521 (PMC5387106; doi:10.3389/fpls.2017.00521)
Supplement: Supplementary Table S2 — Nucleotide (VIGS fragment) identities of TaDIR1 orthologs sequences to BSMV:TaDIR1-2. [file Table2.DOCX]

|  | **Length (bp)** | **Identities** | **Gaps** |
| --- | --- | --- | --- |
| BSMV: TaDIR1-2 | 155 | 155/155(100%) | 0/155 |
| TRIAE_5BS_AA1380450.1 | 155 | 142/155 (93%) | 0/155 |
| TRIAE_5AS_AA1263330.1 | 155 | 138/155 (90%) | 0/155 |
| TRIAE_5AS_AA1263280.1 | 155 | 154/155 (100%) | 0/155 |
| TRIAE_2BL_AA0445540.1 | 155 | 134/155 (86%) | 0/155 |
| TRIAE_2BL_AA0432910.1 | 155 | 133/155 (86%) | 0/155 |
| TRIAE_2BL_AA0435870.1 | 155 | 134/155 (86%) | 0/155 |
| TRIAE_2BL_AA0432920.1 | 155 | 136/155 (88%) | 0/155 |
| TRIAE_2DL_AA0539490.1 | 155 | 134/155 (86%) | 0/155 |
| TRIAE_2AL_AA0305830.1 | 155 | 133/155 (86%) | 0/155 |
| TRIAE_2BL_AA0445100.1 | 155 | 133/155 (86%) | 0/155 |
| TRIAE_2DL_AA0540610.1 | 155 | 135/155 (87%) | 0/155 |
| TRIAE_3AL_AA0648420.1 | 155 | 133/154 (86%) | 0/155 |
| TRIAE_7AS_AA1829590.1 | 155 | 109/155 (57%) | 0/155 |
| TRIAE_7BS_AA1927080.1 | 151 | 110/151 (58%) | 0/151 |
| TRIAE_4BL_AA1037620.1 | 157 | 93/157 (46%) | 6/157 |

**TABLE S2: Nucleotide (VIGS fragment) identities of *TaDIR1* orthologs sequences to BSMV:TaDIR1-2**
