# Supplementary material for: TaDIR1-2, a Wheat Ortholog of Lipid Transfer Protein AtDIR1 Contributes to Negative Regulation of Wheat Resistance against Puccinia striiformis f. sp. tritici
Source: Front Plant Sci. 2017 Apr 11;8:521. doi: 10.3389/fpls.2017.00521 (PMC5387106; doi:10.3389/fpls.2017.00521)
Supplement: Supplementary Table S3 — BSMV: TaDIR1-2 silencing targets predicted by si-Fi analysis. [file Table3.DOCX]

**TABLE S3: BSMV: TaDIR1-2 silencing targets predicted by si-Fi analysis**

**TIGR *Triticum aestivum* transcript assembly release 2**

**BLASTX**

| **Targets** | **Total hits** | **Effi. hits** | **Identity %** | **Gap** | **E-value** | **Score (bit)** |
| --- | --- | --- | --- | --- | --- | --- |
| TRIAE_5AS_AA1263280.1 | 135 | 69 | 93.95 | 0 | 2.00E-136 | 472.3 |
| TRIAE_5BS_AA1380450.1 | 28 | 18 | 92.45 | 0 | 6.00E-127 | 440.6 |
| TRIAE_2DL_AA0539490.1 | 18 | 3 | 86.62 | 0 | 1.30E-81 | 289.9 |
| TRIAE_2AL_AA0305830.1 | 18 | 3 | 86.94 | 0 | 5.20E-84 | 297.8 |
| TRIAE_2BL_AA0435870.1 | 13 | 3 | 86.48 | 0 | 1.30E-81 | 289.9 |
| TRIAE_2BL_AA0445100.1 | 12 | 0 | 86.79 | 0 | 5.20E-84 | 297.8 |
| TRIAE_2BL_AA0432920.1 | 11 | 6 | 88.05 | 0 | 1.50E-93 | 329.6 |
| TRIAE_2BL_AA0445540.1 | 9 | 0 | 89.09 | 0 | 5.40E-87 | 307.8 |
| TRIAE_2DL_AA0540610.1 | 9 | 0 | 86.79 | 0 | 5.20E-84 | 297.8 |
| TRIAE_3AL_AA0648420.1 | 9 | 0 | 88.56 | 0 | 3.20E-82 | 291.9 |
| TRIAE_5AS_AA1263330.1 | 8 | 4 | 90.57 | 0 | 1.00E-112 | 393 |
